# SGLT2 Inhibitors, Functional Capacity, and Quality of Life in Patients With Heart Failure: A Systematic Review and Meta-Analysis

**DOI:** 10.1001/jamanetworkopen.2024.5135

**Published:** 2024-04-04

**Authors:** Michael Gao, Kirtipal Bhatia, Arjun Kapoor, Juan Badimon, Sean P. Pinney, Donna M. Mancini, Carlos G. Santos-Gallego, Anuradha Lala

**Affiliations:** 1 Department of Medicine, Icahn School of Medicine at Mount Sinai, New York, New York; 2 Mount Sinai Fuster Heart Hospital, Mount Sinai Morningside, New York, New York; 3 The Zena and Michael A. Wiener Cardiovascular Institute, Icahn School of Medicine at Mount Sinai, New York, New York; 4 Department of Population Health Science, Icahn School of Medicine at Mount Sinai, New York, New York

## Abstract

**Importance:**

The associations of sodium glucose cotransporter-2 inhibitors (SGLT2is) with reduction in mortality and hospitalization rates in patients with heart failure (HF) are well established. However, their association with improving functional capacity and quality of life (QOL) has been variably studied and less reported.

**Objective:**

To provide evidence on the extent to which SGLT2is are associated with improvement on objective measures of functional capacity and QOL in patients living with HF.

**Data Sources:**

The MEDLINE, EMBASE, and Cochrane databases were systematically searched for relevant articles on July 31, 2023.

**Study Selection:**

Randomized, placebo-controlled clinical trials reporting the effect of SGLT2i on functional outcomes of exercise capacity (peak oxygen consumption [peak VO_2_] or 6-minute walk distance [6MWD]) and/or QOL using validated questionnaires for patients with HF were included.

**Data Extraction and Synthesis:**

Data were extracted by 2 authors following the Preferred Reporting Items for Systematic Reviews and Meta-Analyses 2020 guidelines, and a meta-analysis using the restricted maximum likelihood random-effects model was conducted.

**Main Outcomes and Measures:**

Outcomes of interest included changes in peak VO_2_, 6MWD, and Kansas City Cardiomyopathy Questionnaire-12 total symptom score (KCCQ-TSS), clinical summary score (KCCQ-CSS), and overall summary score (KCCQ-OSS).

**Results:**

In this meta-analysis of 17 studies, 23 523 patients (mean [range] age, 69 [60-75] years) were followed over a period ranging from 12 to 52 weeks. Four studies included peak VO_2_ as an outcome, 7 studies included 6MWD, and 10 studies reported KCCQ scores. Mean (SD) left ventricular ejection fraction was 43.5% (12.4%). Compared with controls, patients receiving SGLT2i treatment experienced significant increases in peak VO_2_ (mean difference [MD], 1.61 mL/kg/min; 95% CI, 0.59-2.63 mL/kg/min; *P* = .002) and 6MWD (MD, 13.09 m; 95% CI, 1.20-24.97 m; *P* = .03). SGLT2i use was associated with increased KCCQ-TSS (MD, 2.28 points; 95% CI, 1.74-2.81 points; *P* < .001), KCCQ-CSS (MD, 2.14 points; 95% CI, 1.53-2.74 points; *P* < .001), and KCCQ-OSS (MD, 1.90 points; 95% CI, 1.41-2.39 points; *P* < .001) scores. Subgroup analysis and meta-regression demonstrated almost all improvements were consistent across ejection fraction, sex, and the presence of diabetes.

**Conclusions and Relevance:**

These findings suggest that in addition to known clinical associations with mortality and hospitalization outcomes, SGLT2i use is associated with improvement in outcomes of interest to patients’ everyday lives as measured by objective assessments of maximal exercise capacity and validated QOL questionnaires, regardless of sex or ejection fraction.

## Key Points

**Question:**

Are sodium glucose cotransporter-2 inhibitors (SGLT2is) associated with functional capacity and quality of life outcomes among patients with heart failure?

**Findings:**

In this systematic review and meta-analysis of 17 studies including 23 523 patients with heart failure, SGLT2is were associated with significant improvements in clinically relevant, patient-centered outcomes including peak oxygen consumption, 6-minute walk distance, and Kansas City Cardiomyopathy Questionnaire-12 scores. Benefits were observed regardless of ejection fraction, sex, or diabetes status.

**Meaning:**

These findings suggest that in addition to its clinical associations with mortality and hospitalization outcomes, SGLT2i therapy is associated with meaningful improvements in patient quality of life, reflected in improved maximal exercise capacity and performance on validated quality of life questionnaires.

## Teaser

This systematic review and meta-analysis assesses associations of sodium glucose cotransporter-2 inhibitors (SGL2i) with functional capacity and quality of life outcomes among patients with heart failure (HF).

## Introduction

More than 64 million people worldwide, including up to 7 million in the US, are living with heart failure (HF).^1,2^ These individuals face not only high rates of hospitalizations and mortality, but also experience substantial limitations in their functional capacity and overall quality of life.^3^ Sodium glucose cotransporter-2 inhibitors (SGLT2is) are the newest class of drugs to be recommended based on the results of several large randomized clinical trials (RCTS) demonstrating reduced cardiovascular mortality and HF hospitalizations with their use.^4,5,6,7^ Perhaps most notably, the SGLT2i class of drugs has been discovered to confer clinical benefit in patients with HF across the spectrum of left ventricular ejection fraction (LVEF), irrespective of whether encountered in the acute or chronic setting.

The data on reduced mortality and hospitalizations are compelling; however, such hard outcomes often do not relay the transformative impact these medications can have on patients’ daily living. Increasingly, patients convey increased interest in feeling better and being able to do more, hoping for tangible improvements in daily life, increased functional capacity, and the ability to pursue activities freely without substantial unfavorable adverse effects. In clinical trial settings, gauging exercise capacity (by walk distance or cardiopulmonary testing) and quantifying quality of life (QOL) using validated instruments is resource intensive.^8^ Furthermore, regulatory bodies and payor models prioritize cost-saving, hard clinical outcomes for drug approval, relegating measures of QOL and functional capacity to be inconsistently measured with varying tools of assessment.^9,10^ As a result, quantifying the benefits an intervention might have on exercise capacity and/or QOL to patients can be challenging. Increased understanding of potential improvement in function may further enhance medication adherence and, thus, lead to greater improvement in outcomes. Addressing this need, we sought to aggregate RCT SGLT2i data to date wherein functional capacity, as measured by peak oxygen consumption (VO_2_), 6-minute walk distance (6MWD), and validated patient-centered QOL surveys, were formally assessed.

## Methods

### Data Sources and Study Selection

The study protocol for this systematic review and meta-analysis was registered a priori in the PROSPERO database. We performed a systematic literature search for all studies analyzing the effect of SGLT2is on objective measures of exercise ability or QOL outcomes measured using validated questionnaires using the Medline, EMBASE, and Cochrane central databases in accordance with the Preferred Reporting Items for Systematic Reviews and Meta-Analyses (PRISMA) reporting guideline.^11^ The systematic review tool Covidence^12^ (Veritas Health Innovation) was used to extract and export studies from all databases and automatically remove duplicate studies.

A detailed explanation of the search terms used for the systematic search is included in the eAppendix in Supplement 1. RCTs published up to July 31, 2023, were considered for inclusion provided they focused exclusively on nonpregnant adults (>18 years) with HF and analyzed the effect of any SGLT2i on 6MWD, peak VO_2_ on cardiopulmonary testing_,_ Kansas City Cardiomyopathy Questionnaire-12 (KCCQ-12) score, or Minnesota Living with Heart Failure Questionnaire (MLHFQ) score compared with a control or placebo group. No language restrictions were applied. Review articles, meta-analyses, abstracts, and presentations at major national cardiovascular meetings were excluded. Secondary or subgroup analyses were excluded unless they provided additional information on outcomes of interest not reported in the primary analysis.

Study selection followed 3 steps. First, 2 authors (M.G. and A.K.) independently screened all study titles and abstracts for potential relevance to the project. The authors (M.G. and A.K.) then conducted a full-text review of all studies remaining after the initial screening for inclusion in the final analysis. Disagreements during screening were resolved either by group consensus or by a third reviewer (K.B.).

### Data Extraction and Compilation

Two authors (M.G. and K.B.) independently extracted and compiled variables and outcomes of interest from eligible studies. For all eligible studies, key study characteristics were extracted, including sample size, study design, SGLT2i used, length of follow-up, baseline medical therapy, and functional outcomes reported by study. Additionally, for each eligible study population, salient baseline and patient characteristics such as age, glomerular filtration rate (GFR), and proportion of participants with diabetes were extracted (eTable 1 in Supplement 1). Outcomes of interest included change in peak VO_2_, 6MWD, KCCQ-12 total symptom score (KCCQ-TSS), KCCQ-12 clinical summary score (KCCQ-CSS), KCCQ-12 overall summary score (KCCQ-OSS), and proportion of study participants with small (≥5 points), moderate (≥10-points), or large (≥15 points) improvement in KCCQ-12 scores at the end of the study interval.

### Quality Assessment

The Risk of Bias 2 (RoB 2) tool^13^ was used to assess the quality of studies considered for final analysis. Based on its risk of bias in 5 domains—randomization and allocation, deviation from intervention, missing participant data, measurement, and selective reporting—2 authors (M.G. and K.B.) independently assigned each included study a bias score of low risk, some concerns, or high risk (eFigure 1 in Supplement 1).

### Statistical Analysis

For continuous outcomes, the adjusted mean difference (MD) between the SGLT2i group and the control group of the trial, reported at the longest duration of follow-up, and the 95% CIs reported by each individual study were used. This represents the most complete adjustment available for baseline covariates. For outcomes where the MD and 95% CI between groups was not homogeneously reported across studies, we utilized the mean values and SD at the end of follow-up to calculate pooled MD and its associated 95% CI. For some studies, SDs were estimated using methods described in the Cochrane handbook (eTable 2 in Supplement 1). Adjusted odds ratios (aORs) with 95% CIs were used to measure the effect size for proportional data. We used the random-effects model to report pooled outcome data.^14^ The restricted maximum likelihood model was used to estimate the between-study variance. Subgroup analysis was conducted to assess the variability of treatment effect across different trial populations. Trials were stratified based on the SGLT2i used, duration of follow-up, and the baseline EF. HF with reduced left ventricular function (HFrEF) was defined as an EF of 40% or less. Significant differences between various subgroups were evaluated using the *Q* statistic.^15^ A random-effects meta-regression analysis was conducted to analyze the interaction of baseline diabetic status and female sex with the primary and secondary outcomes. Heterogeneity among studies was assessed using the Higgins *I^2^* value.^16^ For outcomes of interest with at least 10 included studies, publication bias was assessed using the Egger regression test and through visual inspection of funnel plots to identify asymmetry.^17^ Certainty of assessment was evaluated using the Grading of Recommendations, Assessment, Development, and Evaluations (GRADE) approach.^18^ Statistical analyses were conducted using Stata statistical software version 18 (Stata Corp) using the meta command. A 2-sided *P* value < .05 were considered statistically significant.

## Results

**Figure 1.**
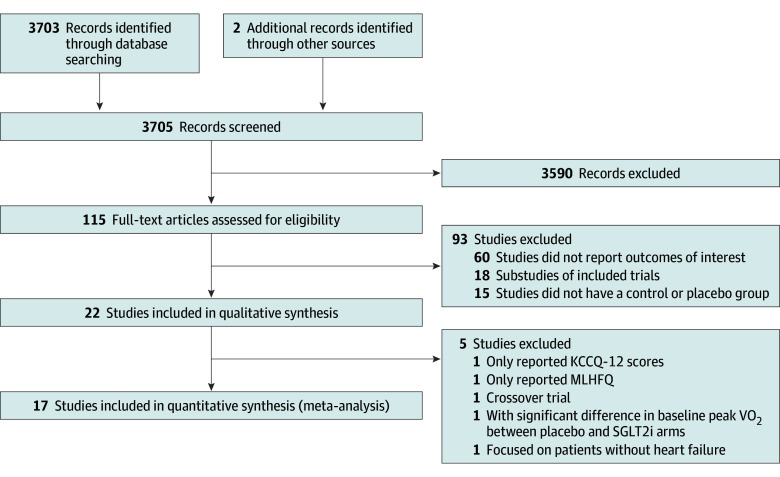
Flow Diagram for Study Identification and Inclusion KCCQ-12 indicates Kansas City Cardiomyopathy Questionnaire-12; MLHFQ, Minnesota Living with Heart Failure Questionnaire; SGLT2i, Sodium glucose cotransporter-2 inhibitor; VO_2_, oxygen uptake.

Our initial systematic database search yielded 3705 studies. After title and abstract review, 115 RCTs were selected for full-text review. While studies considering any cardiac disease were considered during screening, almost all eligible studies which passed full-text review focused on outcomes in patients with HF; the only study to include patients without HF (Verma et al,^19^) was, thus, excluded from final analysis. Ultimately, 17 RCTs^4,5,7,20,21,22,23,24,25,26,27,28,29,30,31,32^ with a total of 23 523 patients were included (Table). A flowchart in Figure 1 details the results of the systematic search and the process of study selection used. Studies excluded during full-text review are listed in eTable 3 in Supplement 1. For all studies, conflicts of interest and trial sponsorships were appropriately disclosed (eTable 2 in Supplement 1).

**Table.** Study Design and Outcomes Among Studies Reporting Functional Outcomes

Source	Study type	SGLT2i used	Participants, No.	Key inclusion criteria	Duration of follow-up	Patient-centered outcomes	Included in the meta-analysis
Nassif et al,^20^ 2019	RCT	Dapagliflozin	263	Chronic heart failure; NYHA class II-III with or without T2D; LVEF ≤ 40%; eGFR ≥ 30 mL/min/1.73 m^2^	3 mo	Mean difference for 6MWD; improvement in KCCQ-CSS and KCCQ-OSS	Yes
Butler et al,^21^ 2020	RCT	Empagliflozin	3705	Chronic heart failure; NYHA class II-IV with or without T2D; LVEF ≤ 40%; eGFR ≥ 20 mL/min/1.73 m^2^	12 mo	Mean difference for KCCQ-TSS, KCCQ-CSS, and KCCQ-OSS; improvement in KCCQ-TSS, KCCQ-CSS, and KCCQ-OSS	Yes
McMurray et al,^5^ 2020	RCT	Dapagliflozin	4443	Chronic heart failure; NYHA class II-IV with or without T2D; LVEF ≤ 40%; eGFR ≥ 30 mL/min/1.73 m^2^	8 mo	Improvement in KCCQ-TSS, KCCQ-CSS, and KCCQ-OSS	Yes
Carbone et al,^22^ 2020	RCT^a^	Canagliflozin	36	Chronic heart failure; NYHA class II-IV with T2D; LVEF ≤ 40%; eGFR ≥ 50 mL/min/1.73 m^2^	3 mo	Mean difference for peak VO_2_ and MLHFQ	Yes
Lee et al,^23^ 2021	RCT	Empagliflozin	105	Chronic heart failure; NYHA class II-IV; LVEF ≤ 40%; eGFR ≥ 30 mL/min/1.73 m^2^	9 mo	Mean difference for KCCQ-TSS and 6MWD	Yes
Bhatt et al,^33^ 2020	RCT	Sotagliflozin	1222	Acute heart failure irrespective of LVEF with T2D; eGFR ≥ 30 mL/min/1.73 m^2^	9 mo	Mean difference for KCCQ-12	No
Tanaka et al,^45^ 2020	RCT^b^	Canagliflozin	233	Chronic heart failure irrespective of LVEF; NYHA class I-III with T2D; eGFR ≥ 45 mL/min/1.73 m^2^	6 mo	Mean difference for MLHFQ	No
EMPERIAL-REDUCED,^24^ 2021	RCT	Empagliflozin	312	Chronic heart failure; NYHA II-IV with or without T2D; LVEF ≤ 40%; eGFR ≥ 20 mL/min/1.73 m^2^	3 mo	Mean difference for KCCQ-TSS, KCCQ-CSS, KCCQ-OSS, and 6MWD; improvement in KCCQ-TSS	Yes
Abraham et al,^24^ 2021	RCT	Empagliflozin	315	Chronic heart failure; NYHA class II-IV with or without T2D; LVEF > 40%; eGFR ≥ 20 mL/min/1.73 m^2^	3 mo	Mean difference for KCCQ-TSS, KCCQ-CSS, KCCQ-OSS, and 6MWD; improvement in KCCQ-TSS	Yes
Jensen et al,^25^ 2021	RCT	Empagliflozin	190	Chronic heart failure; NYHA class I-III with or without T2D; LVEF ≤ 40% and eGFR ≥ 30 mL/min/1.73 m^2^	3 mo	Mean difference for KCCQ-CSS, KCCQ-OSS, and KCCQ-TSS	Yes
Anker et al,^4^ 2021	RCT	Empagliflozin	5988	Chronic heart failure; NYHA class II-IV with or without T2D; LVEF ≥ 40%; eGFR ≥ 20 mL/min/1.73 m^2^	12 mo	Mean difference for KCCQ-TSS, KCCQ-CSS, and KCCQ-OSS; improvement in KCCQ-TSS, KCCQ-CSS, and KCCQ-OSS	Yes
Nassif et al,^26^ 2021	RCT	Dapagliflozin	65	Chronic heart failure irrespective of LVEF; NYHA class II-IV with or without T2D; prior CardioMEMS device; eGFR ≥ 30 mL/min/1.73 m^2^	3 mo	Improvement in KCCQ-CSS and KCCQ-OSS	Yes
Nassif et al,^27^ 2021	RCT	Dapagliflozin	324	Chronic heart failure; NYHA class II-IV with or without T2D; LVEF ≥ 45%; eGFR ≥ 20 mL/min/1.73 m^2^	3 mo	Mean difference for KCCQ-CSS, KCCQ-OSS, KCCQ-TSS, and 6MWD; Improvement in KCCQ-CSS, and KCCQ-TSS	Yes
Santos-Gallego et al,^28^ 2021	RCT	Empagliflozin	84	Chronic heart failure; NYHA class II- III class without T2D; LVEF ≤ 50%; GFR < 30 mL/kg/min	6 mo	Mean difference for KCCQ-12, peak VO_2_, and 6MWD	Yes
Spertus JA et al,^29^ 2022	RCT	Canagliflozin	476	Chronic heart failure irrespective of LVEF; eGFR ≥ 30 mL/min/1.73 m^2^	3 mo	Mean difference for KCCQ-CSS, KCCQ-OSS, and KCCQ-TSS	Yes
Ilyas et al,^46^ 2022	RCT	Dapagliflozin	19	Chronic heart failure with T2D; LVEF ≤ 35%.	0.5 mo	6MWD (cross over trial and reported median and IQR)	No
Voors et al,^30^ 2022	RCT	Empagliflozin	495	Acute heart failure; NYHA class II-IV with or without T2D; eGFR ≥ 20 mL/min/1.73 m^2^	3 mo	Mean difference for KCCQ-CSS, KCCQ-OSS, and KCCQ-TSS	Yes
Palau et al,^31^ 2022	RCT	Dapagliflozin	90	Chronic heart failure; NYHA class II-IV; LVEF ≤ 40%; eGFR ≥ 30 mL/min/1.73 m^2^	3 mo	Mean difference for peak VO_2_, 6MWD, and MLHFQ	Yes
Reis et al,^32^ 2022	RCT	Dapagliflozin	40	Chronic heart failure; NYHA class II-IV without T2D; LVEF < 50%; GFR ≥ 30 mL/min	6 mo	Mean difference for peak VO_2_	Yes
Solomon et al,^7^ 2022	RCT	Dapagliflozin	6263	Chronic heart failure, NYHA class II-IV with or without T2D; LVEF ≥ 40%; eGFR ≥ 25 mL/min/1.73 m^2^	8 mo	Mean difference for KCCQ-TSS, KCCQ-CSS, and KCCQ-OS; improvement in KCCQ-TSS, KCCQ-CSS, and KCCQ-OSS	Yes
Hundertmark et al,^34^ 2023	RCT	Empagliflozin	72	Chronic heart failure irrespective of LVEF; NYHA class II-IV with and without T2D; eGFR ≥ 30 mL/min/1.73 m^2^	3 mo	Mean difference for peak VO_2_ (Peak VO_2_ levels at baseline were significantly different between the placebo and SGLT2i groups which precluded inclusion of trial in the meta-analysis)	No

Abbreviations: 6MWD, 6-minute walk distance (meters); eGFR, estimated glomerular filtration rate; KCCQ-CSS; Kansas City Cardiomyopathy Questionnaire-Clinical Summary Score; KCCQ-OSS, Kansas City Cardiomyopathy Questionnaire-Overall Summary Score; KCCQ-TSS, Kansas City Cardiomyopathy Questionnaire-Total Symptom Score; LVEF, left ventricular ejection fraction; MLHFQ, Minnesota Living with Heart Failure Questionnaire; NYHA, New York Heart Association; RCT, randomized clinical trial; SGLT2i, Sodium glucose cotransporter-2 inhibitor; T2D, diabetes; VO_2_, oxygen consumption.

^a^ Control group received sitagliptin.

^b^ Control group received glimepiride.

Studies evaluating only MLHFQ scores (3 studies) or KCCQ-12 scores (2 studies) as outcomes were excluded due to the low number of total trials and the inability to effectively pool trial data for statistical analysis due to significant heterogeneity in data reporting between studies. To this effect, the recent landmark SOLOIST trial by Bhatt et al^33^ studying the effect of sotagliflozin on cardiovascular end points was excluded because it did not include relevant study end points (6MWD or peak VO2) in its analysis.

### Study Characteristics

Of the 23 523 participants (mean [range] age, 69 [60-75 years]; 8472 female [36%]), 10 887 (46%) had type 2 diabetes. Patients were followed over a period ranging from 12 to 52 weeks. The mean (SD) GFR was 61.9 (3.5) mL/min/1.73 m^2^ and mean (SD) N-terminal pro–B-type natriuretic peptide was 1380 (511) pg/mL (to convert to nanograms per liter, multiply by 1.0) for all participants (eTable 1 in Supplement 1). Mean (SD) LVEF was 43.5% (12.4%). Of the 17 trials, 10 included patients with HFrEF,^5,6,20,22,23,24,25,28,31,32^ 4 had patients with HF with preserved EF (HFpEF),^4,7,24,27^ and 3 had patients with both HFrEF and HFpEF.^26,29,30^

### Intervention Characteristics

Empagliflozin was the SGLT2i examined in 9 studies,^4,6,23,24,25,26,28,30^ dapagliflozin in 6 studies,^5,7,20,27,31,32^ and canagliflozin in 2 studies.^22,29^ As mentioned previously, studies including sotagliflozin could not be included due to end points analyzed. Salient baseline and population characteristics of each study cohort are detailed in eTable 1 in Supplement 1.

### Exercise Capacity Analysis

**Figure 2.**
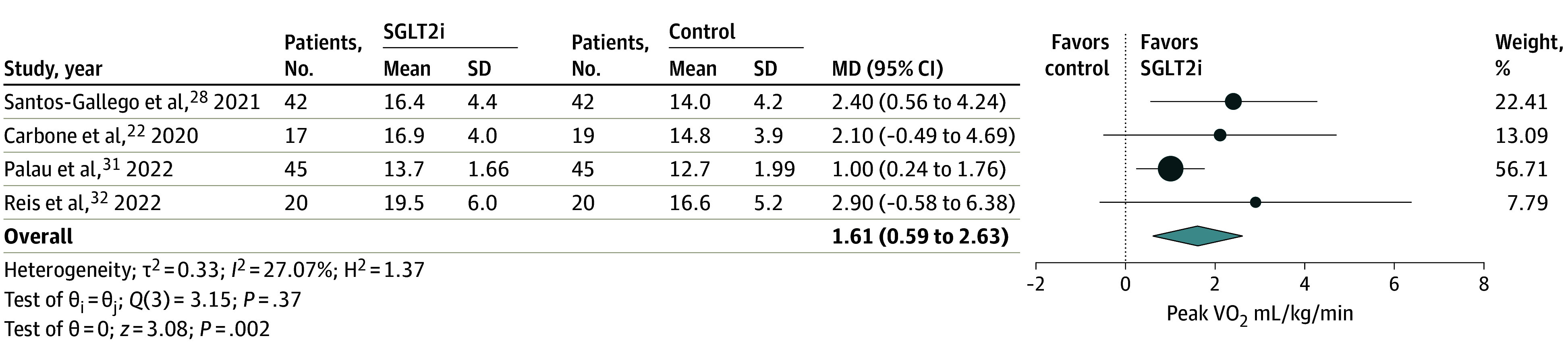
Association of SGLT2i Treatment With Peak Oxygen Consumption Peak oxygen consumption (VO_2_) was measured as milliliters per kilogram per minute. MD indicates mean difference; SGLT2i, sodium glucose cotransporter-2 inhibitor.

**Figure 3.**
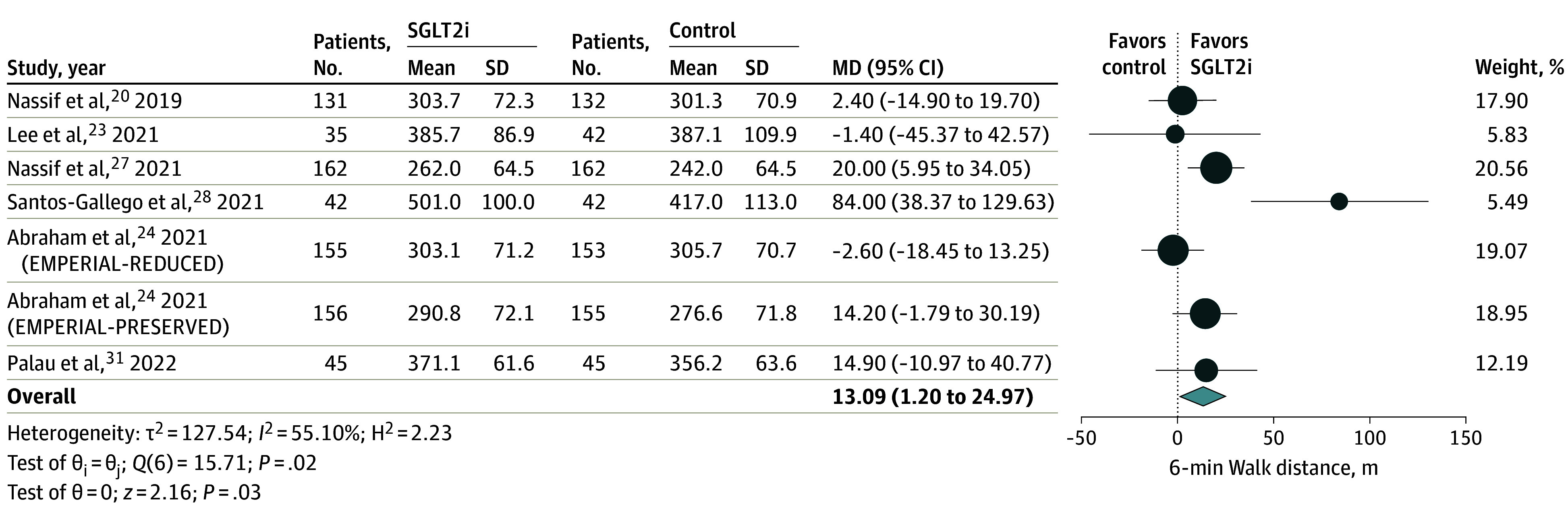
Association of SGLT2i Treatment With 6-Minute Walk Distance The 6-minute walk distance was measured in meters. MD indicates mean difference; SGLT2i, sodium glucose cotransporter-2 inhibitor.

Four studies^22,28,31,32^ including 250 patients with HFrEF evaluated peak VO_2_ (Figure 2) with a follow-up time ranging between 3 to 6 months. SGLT2i therapy was associated with significant improvement in peak VO_2_ compared with placebo (MD, 1.61 mL/kg/min; 95% CI, 0.59-2.63 mL/kg/min; *P* = .002). Heterogeneity between studies was moderate. Seven trials^20,23,24,26,28,31^ including 1457 patients evaluated 6MWD (Figure 3). Follow-up duration varied between 3 to 6 months. In pooled analysis, patients treated with SGLT2is were able to walk over 13 more meters compared with the control group (MD, 13.09 m; 95% CI; 1.20-24.97 m; *P* = .03). No significant subgroup interaction for outcomes by baseline LVEF (*P *for interaction = .88), SGLT2i used (*P *for interaction = .70), or follow-up time (*P *for interaction = .47) was observed (eFigure 2 in Supplement 1). The difference in proportions of patients with type 2 diabetes included in the trials explained most of the heterogeneity (eFigure 3 in Supplement 1). Because our study used mean values for peak VO_2_ and 6MWD at the end of trial follow-up to estimate pooled MD across trials, only studies where baseline peak VO_2_ and 6 MWD were similar between the SGLT2i group and placebo group were included, resulting in the exclusion of the EMPA-VISION trial by Hundertmark et al^34^ (Figure 1). This conservative measure was taken to ensure that any improvement noted for patients treated with SGLT2is likely represented a true therapeutic benefit. See eTable 4 in Supplement 1 for more data on 6MWD.

### QOL Analysis

**Figure 4.**
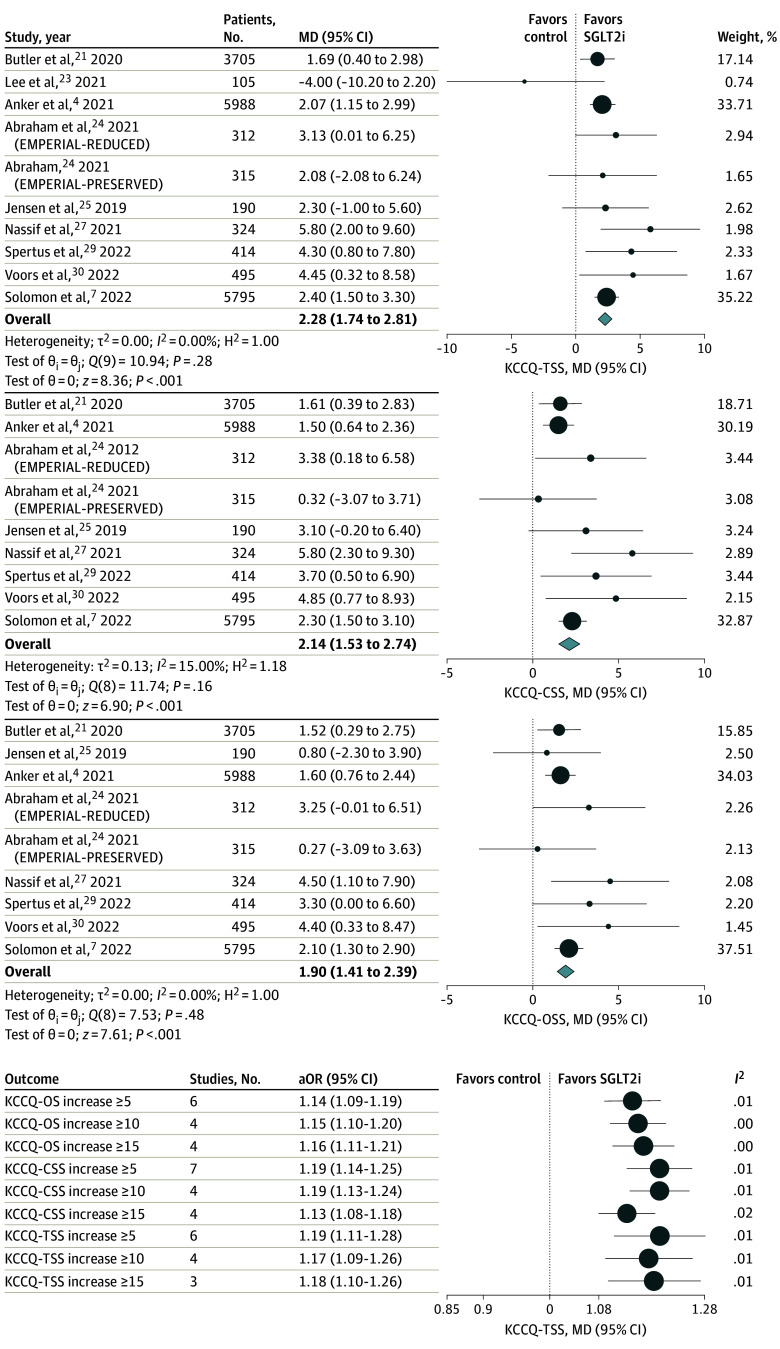
Association of SGLT2i Treatment With Kansas City Cardiomyopathy Questionnaire-12 (KCCQ-12) Outcomes CSS indicates clinical summary score; MD, mean difference; OSS, overall summary score; SGLT2i, sodium glucose cotransporter-2 inhibitor; TSS; total summary score.

Ten studies^4,6,7,23,24,25,27,29,30^ including 17 703 patients evaluated KCCQ-TSS scores and 9 studies^4,6,7,24,25,27,29,30^ including 17 598 patients evaluated both KCCQ-CSS and KCCQ-OSS scores. Follow-up time varied between 3 months to 1 year (Figure 4). Patients taking an SGLT2i had a significantly higher mean change in KCCQ-TSS score (MD, 2.28; 95% CI, 1.74-2.81 points; *P* < .001), KCCQ-OSS score (MD, 1.90 points; 95% CI, 1.41-2.39 points; *P* < .001), and KCCQ-CSS score (MD, 2.14 points; 95% CI, 1.53-2.74 points; *P* < .001) compared with those receiving placebo (Figure 4). There was no significant heterogeneity in outcomes for KCCQ-TSS scores, KCCQ-OSS scores, and KCCQ-CSS scores. Likewise, there was no significant subgroup interaction for KCCQ-12 outcomes by either baseline LVEF, SGLT2i used, or follow-up time (eFigures 4-6 in Supplement 1). Grouped differently, patients treated with SGLT2is had a 19% higher likelihood of experiencing a small but clinically significant (>5 points) improvement in their KCCQ-TSS score (aOR, 1.19; 95% CI, 1.11-1.28), 14% higher likelihood of experiencing a small but clinically significant (>5 points) improvement in their KCCQ-OSS score (aOR, 1.14; 95% CI, 1.09-1.19), and 19% higher likelihood of experiencing a small but clinically significant (>5 points) improvement in their KCCQ-CSS score (aOR, 1.19; 95% CI, 1.13-1.25) (Figure 4). For all levels of improvement in KCCQ scores, no significant interstudy heterogeneity was observed (eFigure 7 in Supplement 1).

Meta-regression analysis revealed that most improvements in functional capacity outcomes seen with SGLT2i use were not associated with sex or baseline diabetic status. However, an inverse association between proportion of patients with type 2 diabetes type and improvement in 6MWD with SGLT2i treatment was observed. Because only 4 trials^22,28,31,32^ evaluated peak VO_2_, no subgroup or meta-regression analysis were performed (eFigure 8 in Supplement 1).

### Evaluation of Risk of Study Bias and Certainty of Evidence of Included Studies

All publications were judged to have low or intermediate risk of bias using the RoB 2 tool. All publications were judged to have high or moderate certainty of evidence using the GRADE approach (eFigure 1 in Supplement 1).

### Evaluation of Asymmetry of Evidence

KCCQ-TSS was the only outcome of interest that included at least 10 studies to allow for funnel plot analysis. No publication bias was revealed (eFigure 9 in Supplement 1).

## Discussion

The findings of our systematic review and meta-analysis suggest that while the benefits of SGLT2is on mortality and HF hospitalizations have been established across the spectrum of LVEF, and irrespective of the acuity and severity of HF, their effect on functional capacity and QOL remained incompletely and inconsistently captured.^8^ In this integrated analysis of 17 trials including more than 23 000 patients, we demonstrated an association of SGLT2i treatment with significantly higher performance on all 5 functional capacity and QOL end points evaluated (peak VO_2_, 6MWD, KCCQ-TSS, KCCQ-OS, and KCCQ-CSS), all of which can be more readily communicated to patients. Our meta-analysis represents, to our knowledge, the most systematic effort to evaluate the associations of SGLT2is with functional capacity and QOL outcomes to date, incorporating both clinician-measured objective metrics (6MWD and peak VO_2_) and patient-reported (KCCQ-12 scores) metrics.

Treatment with SGLT2is was associated with a modest increase (2 points) in KCCQ-12 scores; as such, patients randomized to SGLT2i therapy were approximately 15% to 20% more likely to experience a clinically significant improvement (of at least 5, 10, or 15 points) in KCCQ-12 scores across all domains. The observed improvements in KCCQ-12 scores were also consistent across included trials. There was significant improvement in the treatment group in 6 of 9 trials^4,6,7,27,29,30^ for KCCQ-OSS, 7 of 9 trials for KCCQ-CSS,^4,6,7,24,27,29,30^ and 7 of 9 trials for KCCQ-TSS^4,6,7,24,27,29,30^. These pooled data help contextualize a prior meta-analysis in 2022 by Yang et al^35^ of only patients with HFpEF in which numerical values of KCCQ-12 score improvement were similar but did not reach statistical significance. Though more heterogenous, we also found patients treated with SGLT2i therapy experienced significantly increased 6MWD and peak VO_2_ compared with their untreated counterparts. An approximately 13-meter improvement in 6MWD with SGLT2i was observed, dispelling early concerns following the EMPERIAL trial by Mancini et al,^36^ which showed neutral effects on exercise capacity. Further corroborating the positive effect of SGLT2is on functional capacity is the observation of an increase in peak VO2 by 1.61 mL/kg/min in treated patients, a benchmark and objective functional parameter that predicts mortality in HF.^36^ These readily translatable results help inform shared discussions in clinical settings where SLGT2is are introduced and reinforced for patients with HF.

### Mechanistic Considerations

The mechanism by which SGLT2is may improve functional capacity is complex and multifactorial. In addition to potential, direct cardiomechanical effects such as reversing ventricular remodeling^28^ and reducing interstitial cardiac fibrosis,^37,38^ SGLT2is enhance the efficiency of cardiac and skeletal muscle metabolism by promoting ketogenesis and a metabolic shift toward ketone-body utilization.^39^ SGLT2is also improve iron deficiency in patients with HF by reducing hepcidin levels and, thus, increase iron mobilization and intestinal iron absorption.^40^ Because HF is commonly associated with low tissue iron levels, this effect enhances oxygen-carrying capacity and reverses mitochondrial dysfunction and impaired energetics in these patients. Notably, a recent subanalysis of the EMPATROPISM trial by Angermann et al^41^ demonstrated that the increase in cardiac iron content in patients treated with empagliflozin was associated with improvements in peak VO2 and 6MWD. Moreover, SGLT2is prevent HF-induced skeletal muscle remodeling and have been observed to possibly even reverse muscular atrophy.^42,43^

### Heterogeneity

Heterogeneity of outcomes between studies for all outcomes was either low or moderate. The moderate heterogeneity for the 6MWD was predominantly explained by differences in the proportion of patients with diabetes included in each study; meta-regression analysis demonstrated studies with a lower proportion of patients with underlying diabetes had a higher mean increase in 6MWD at the end of follow-up. This discrepancy could be explained by confounding musculoskeletal and neurological comorbidities in patients with diabetes, such as peripheral neuropathy, that attenuate the improvement in 6MWD observed with SGLT2is.^44^ Nevertheless, for all other metrics, sensitivity analysis and meta-regression demonstrated the benefits of SGLT2i treatment for improving functional capacity occurred independent of patient characteristics such as LVEF, sex, or diabetic status. As physicians, this finding gives us confidence that we can equitably use SGLT2is to improve the QOL across a diverse segment of patients.

Most meta-analyses of SGLT2is have focused on the effects of mitigating cardiovascular and renal events including mortality, myocardial infarction, hospitalization for HF, as well progression of kidney disease. While such outcomes are vital, they do not reflect the beneficial impact on patients’ daily lives. For patients with HF in particular, poor exercise intolerance can result in crippling debilitation, rendering limitations in being able to participate in exercise training or targeted physical therapy that leads to a downward spiral of worsening physical limitation. Thus, being able to relay an expected 13-meter improvement in 6MWD, for example, could mean the difference between a patient confined to their room and a patient who can answer the door or reach the bathroom comfortably. Herein, one can emphasize the utility of SGLT inhibitors, not only for improving survival among patients with HF, but also to make daily lives more tenable and potentially fulfilling.

### Limitations

Our study has certain limitations. We analyzed outcomes at the study level and lacked individual patient data, which would allow for more standardized end point assessments, such as the net improvement in 6MWD and peak VO_2_. A patient-level analysis would have also allowed for the inclusion of the Hundertmark et al,^34^ which was excluded from the analysis due to significant difference in baseline peak VO_2_ values between placebo and SGLT2i groups. These patient-specific data could further clarify the effects of diabetes and sex on SGLT2i therapy, refining subgroup analyses. Limited trials investigating peak VO_2_ potentially impacted our conclusions. However, the improvement in peak VO_2_ correlated well with the level of improvement seen with 6MWD and is a associated with survival in precardiac transplantation.^36^ Finally, the potential effect of sotagliflozin was not included in this study due to a lack of functional capacity assessment and early termination, making an understanding of sotagliflozin-specific effect on QOL outcomes less robust.^45^

## Conclusions

In this meta-analysis, regardless of sex, diabetic status, or EF, patients receiving SGLT2i therapy for HF experienced significant improvements in patient-centered metrics of functional capacity and QOL as measured by peak VO_2_, 6MWD, and KCCQ-12 scores. These findings suggest that SGLT2i therapies should be considered for improving functional capacity and QOL in addition to reducing the risk of hospitalization and mortality among patients with HF.

## Notes

Supplement 1. **eAppendix.** Search Strategy **eTable 1.** Baseline Characteristics of Included Studies **eFigure 1.** Risk of Bias and Certainty of Evidence Summary **eTable 2.** Conflict of Interest Table for the 17 Included Studies **eTable 3.** Table of Excluded Studies After Full-Text Review **eFigure 2.** Subgroup Analyses: 6-MWD **eFigure 3.** Bubble Plots by Proportion of Females and Diabetic Patients Included in Trials Reporting 6-MWD **eTable 4.** Studies that Reported Means and 95% Confidence Intervals for the 6-MWD at the End of Follow-Up Used to Derive Standard Deviations **eFigure 4.** Subgroup Analyses: KCCQ-TSS **eFigure 5.** Subgroup Analyses: KCCQ-OS **eFigure 6.** Subgroup Analyses: KCCQ-CSS **eFigure 7.** Pooled Results of Improvement in KCCQ Scores **eFigure 8.** Bubble Plots by Proportion of Females and Diabetic Patients Included in Trials Reporting KCCQ-TSS, KCCQ-OS, and KCCQ-CSS **eFigure 9.** Funnel Plot: KCCQ-TSS

Supplement 2.
Data Sharing Statement
