# Supplementary material for: SGLT2 Inhibitors, Functional Capacity, and Quality of Life in Patients With Heart Failure: A Systematic Review and Meta-Analysis
Source: JAMA Netw Open. 2024 Apr 4;7(4):e245135. doi: 10.1001/jamanetworkopen.2024.5135 (PMC11192183; doi:10.1001/jamanetworkopen.2024.5135)
Supplement: Supplement 1. — eAppendix. Search Strategy eTable 1. Baseline Characteristics of Included Studies eFigure 1. Risk of Bias and Certainty of Evidence Summary eTable 2. Conflict of Interest Table for the 17 Included Studies eTable 3. Table of Excluded Studies After Full-Text Review eFigure 2. Subgroup Analyses: 6-MWD eFigure 3. Bubble Plots by Proportion of Females and Diabetic Patients Included in Trials Reporting 6-MWD eTable 4. Studies that Reported Means and 95% Confidence Intervals for the 6-MWD at the End of Follow-Up Used to Derive Standard Deviations eFigure 4. Subgroup Analyses: KCCQ-TSS eFigure 5. Subgroup Analyses: KCCQ-OS eFigure 6. Subgroup Analyses: KCCQ-CSS eFigure 7. Pooled Results of Improvement in KCCQ Scores eFigure 8. Bubble Plots by Proportion of Females and Diabetic Patients Included in Trials Reporting KCCQ-TSS, KCCQ-OS, and KCCQ-CSS eFigure 9. Funnel Plot: KCCQ-TSS [file jamanetwopen-e245135-s001.pdf]

Supplemental Online Content

Gao M, Bhatia K, Kapoor A, et al. SGLT2 Inhibitors, functional capacity, and quality of life in patients with heart failure. *JAMA Netw Open.* 2024;7(4):e245135. doi:10.1001/jamanetworkopen.2024.5135

eAppendix. Search Strategy

eTable 1. Baseline Characteristics of Included Studies

eFigure 1. Risk of Bias and Certainty of Evidence Summary

eTable 2. Conflict of Interest Table for the 17 Included Studies

eTable 3. Table of Excluded Studies After Full-Text Review

eFigure 2. Subgroup Analyses: 6-MWD

eFigure 3. Bubble Plots by Proportion of Females and Diabetic Patients Included in Trials Reporting 6-MWD

eTable 4. Studies that Reported Means and 95% Confidence Intervals for the 6-MWD at the End of Follow-Up Used to Derive Standard Deviations

eFigure 4. Subgroup Analyses: KCCQ-TSS

eFigure 5. Subgroup Analyses: KCCQ-OS

eFigure 6. Subgroup Analyses:KCCQ-CSS

eFigure 7. Pooled Results of Improvement in KCCQ Scores

eFigure 8. Bubble Plots by Proportion of Females and Diabetic Patients Included in Trials Reporting KCCQ-TSS, KCCQ-OS, and KCCQ-CSS

eFigure 9. Funnel Plot: KCCQ-TSS

This supplemental material has been provided by the authors to give readers additional information about their work.

© 2024 Gao M et al. *JAMA Network Open.*

eAppendix. Search Strategy

#1 (sodium-glucose transporter 2 inhibitors) OR (sodium glucose co-transporter type 2 inhibitor)) OR (sodium glucose cotransporter type 2 inhibitor)) OR (sodium glucose co- transporter 2 inhibitor)) OR (sodium glucose cotransporter 2 inhibitor)) OR (sodium dependent glucose co-transporter 2 inhibitor)) OR (sodium dependent glucose cotransporter 2 inhibitor)) OR (sodium dependent glucose transporter 2 inhibitor)) OR (sodium glucose linked cotransporter 2 inhibitor)) OR (sodium glucose linked transporter 2 inhibitor)) OR (SGLT-2 inhibitor)) OR (inhibitor, SGLT2)) OR (Gliflozins)) OR (Gliflozin)) OR (Dapagliflozin)) OR (Empagliflozin)) OR (Canagliflozin)) OR (Ipragliflozin)) OR (Luseogliflozin)) OR (Tofogliflozin)) OR (Ertugliflozin)) OR (Sotagliflozin)

#2 (((((6-minute walk test) OR (six-minute walk test)) OR (6 minute walk test)) OR (6-MWT)) OR (6MWT)) OR (6-min walk test)

#3 ((KCCQ) OR (KCCQ-12)) OR (Kansas City Cardiomyopathy Questionnaire)

#4 accelerometer OR peak oxygen consumption OR cardiopulmonary exercise test OR Peak VO2 OR CPET

#5 minnesota living heart failure questionnaire OR mlhfq OR chronic heart failure questionnaire OR chfq

#6 exercise ability OR exercise capacity OR quality of life OR symptoms OR functional capacity OR physical function OR Health-Related Quality of Life OR quality of life OR QoL

#7. #6 OR #5 OR #4 OR #3 OR #2

#8. Search: #7 AND #1

© 2024 Gao M et al. *JAMA Network Open.*

eTable 1. Baseline Characteristics of Included Studies

Study/ Year Published	Age (yrs)	Females (%)	DM (%)	GFR (ml/kg/ 1.73m^2)	BMI (kg/m^2)	LVEF, (%)	B-blocker, (%)	ACE-I, (%)	ARNI, (%)	MRA, (%)	ARB, (%)	NYHA class II	NYHA class III-IV	NT-BNP, (pg/ml)
**DEFINE-HF 2019**[20]	62.<br>60	27.5<br>25.8	61.8<br>64.4	66.9<br>71.2	30.7<br>30.6	27.2<br>25.2	99.2<br>93.3	58<br>60.6*	35.9<br>20.8	58<br>63.6	58<br>60.6*	69.5<br>62.1	30.5<br>37.9	1136<br>1136
**EMPEROR REDUCED 2020**[21]	67.2<br>66.5	23.5<br>24.4	49.8<br>49.8	61.8<br>62.2	28<br>27.8	27.7<br>27.2	94.7<br>94.7	88.8<br>89.6*	18.3<br>20.7	70.1<br>72.6	88.8<br>89.6*	75<br>75	24.9<br>30	1887<br>1926
**DAPA-HF 2020**[5]	66.2<br>66.5	23.8<br>23.0	41.8<br>41.8	66.0<br>65.5	28.2<br>28.1	31.2<br>30.9	96.0<br>96.2	56.1<br>56.1	10.5’<br>10.9	71.5<br>70.6	28.4<br>26.7	67.7<br>67.4	32.3<br>32.7	1428<br>1446
**EMPERIAL-REDUCED 2021**[24]	69<br>70	22.4<br>28.8	55.8<br>64.1	56.8<br>53	29.2<br>30	30<br>30	94.9<br>94.2	51.9<br>55*	39.1<br>34	60.9<br>55.8	51.9<br>55*	64.7<br>64.7	35.3<br>35.3	1458<br>1559
**EMPERIAL PRESERVED 2021**[24]	74<br>75	44.6<br>41.8	54.8<br>47.5	54.5<br>58.5	30.1<br>28.8	53<br>53	89.2<br>89.2	73.9<br>75.2*	3.2<br>3.8	35<br>31.6	73.9<br>75.2*	74.5<br>79.7	24.8.<br>20.3	966<br>843
**CANA-HF 2020**[22]	58<br>54.3	23.5<br>21.1	100<br>100	74.7<br>83.3	34.5<br>38.8	31.6<br>27	94.1<br>94.7	82.4<br>57.9*	11.8<br>21.1	52.9<br>68.4	82.4<br>57.9*	58.8<br>63.2	41.2<br>36.8	243<br>492

© 2024 Gao M et al. *JAMA Network Open.*

**SUGAR-DM-HF 2020[23]**	68.2<br>69.2	65.4<br>81.1	76.9<br>79.2	69.5<br>65.1	30.9<br>30.4	NA	88.5<br>94.3	48.1<br>45.3	40.4<br>28.3	61.5<br>58.5	5.8<br>22.6	71.2<br>83	28.8<br>17	475<br>466
**EMPIRE-HF 2021[25]**	64<br>63	17<br>13	20<br>15	73<br>74	29<br>29	30<br>30	96<br>94	95<br>97*	33<br>28	65<br>66	95<br>97*	76<br>81	19<br>12	582<br>605
**EMPEROR PRESERVED 2021[4]**	71.8<br>71.9	44.6<br>44.7	49.2<br>49.8	60.6<br>60.6	29.77<br>29.9	54.3<br>54.3	86.7<br>85.9	81<br>80.4*	2.2<br>2.3	37.3<br>37.6	81<br>80.4*	81.1<br>81.9	18.5<br>18.1	994<br>946
**EMBRACE-HF 2021[26]**	69.5<br>62.9	36.4<br>37.5	54.5<br>50	51.2<br>62.7	33.5<br>33.8	46.7<br>40.7	87.9<br>90.6	33.3<br>25*	39.4<br>53.1	27.3<br>62.5	33.3<br>25*	42.4<br>50	54.5<br>50	865.5<br>563.5
**PRESERVED HF 2021[27]**	69<br>71	56.8<br>56.8	55.6<br>56.2	56<br>54	35.1<br>34.6	60<br>60	73.5<br>71.6	60.5<br>60.5*	1.2<br>1.9	30.9<br>42	60.5<br>60.5*	59.3<br>55.6	40.1<br>44.4	641<br>710
**EMPA-TROPISM 2021[28]**	64.2<br>59.9	36<br>36	0<br>0	80<br>83	29.3<br>30	36.2<br>36.5	86<br>90	38<br>45*	50<br>36	31<br>36	38<br>45*	N/R	N/R	N/R
**CHIEF-HF 2022[29]**	62.9<br>64	46.8<br>42.4	29.7<br>26.1	N/R	N/R	NA	N/R	NA	N/R	N/R	NA	N/R	N/R	NA
**EMPULSE 2022[30]**	71<br>70	32.5<br>35.1	46.3<br>43.8	50<br>54	28.35<br>29.08	31<br>32	80.4<br>78.5	33.2<br>33.6	13.6<br>17.0	57<br>47.2	24.2<br>19.6	35.8<br>34.3	60.4<br>63.4	3299<br>3106

© 2024 Gao M et al. *JAMA Network Open.*

**DAPA-VO2 2022**[31]	69.8<br>67.3	22.2<br>24.4	35.6<br>28.9	64.1<br>69.4	27.3<br>28.1	33.7<br>34	91.1<br>91.1	97.8<br>95.6*	88.9<br>88.9	77.8<br>77.1	97.8<br>95.6*	NA	NA	1085<br>1620
**Reis et al. 2022**[32]	60.3<br>61.7	15<br>20	0<br>0	68.7<br>72.5	NA	34.5<br>33.5	100<br>95	100<br>100*	NA	95<br>85	100<br>100*	70<br>90	30<br>10	890.5<br>747.7
**DELIVER 2022**[7]	71.8<br>71.5	43.6<br>44.2	44.7<br>44.9	61<br>61	29.8<br>29.9	54<br>54.3	82.8<br>82.5	36.5<br>36.7	5.3<br>4.3	42.8<br>42.4	36.2<br>36.4	73.9<br>76.6	31.3<br>23.4	NA<br>NA

© 2024 Gao M et al. *JAMA Network Open.*

eFigure 1. Risk of Bias and Certainty of Evidence Summary TableA. ROB 2.0 Risk of Bias Table

Study	Bias from Randomization Errors	Bias due to Deviation from Intended Intervention	Bias from Missing Data	Measurement Bias	Bias from Selective Reporting	Overall Bias
EMPULSE 2022	+	+	+	+	+	+
EMPA-TROPISM 2021	+	+	+	+	+	+
EMPEROR-REDUCED 2020	+	+	+	+	-	-
EMPEROR-PRESERVED 2020	+	+	+	+	-	-
SUGAR-DM-HF 2020	+	+	+	+	+	+
CHIEF-HF 2022	+	+	+	+	+	+
EMPERIAL-REDUCED 2021	+	+	+	+	-	-
EMPERIAL-PRESERVED 2021	+	+	+	+	-	-
DAPA-HF 2019	+	+	+	+	+	+
EMPIRE-HF 2020	+	+	+	+	+	+
DELIVER 2022	+	+	+	+	-	-
EMBRACE-HF 2021	+	+	+	+	+	+
DEFINE-HF 2019	+	+	+	+	+	+
DAPA-VO2 2022	+	+	+	+	+	+
PRESERVED-HF 2021	+	+	+	+	+	+
CANA-HF 2020	+	+	+	+	+	+
Reis et al. 2022	+	+	+	+	+	+

+	Low Risk
-	Some Concerns
X	High Risk

© 2024 Gao M et al. *JAMA Network Open.*

B. Grade Assessment of Certainty

Study	GRADE Assessment of Certainty
EMPULSE 2022	High (low bias and inconsistency, RCT design)
EMPA-TROPISM 2021	High (low bias and inconsistency, RCT design)
EMPEROR-REDUCED 2020	High (low bias and inconsistency, RCT design)
EMPEROR-PRESERVED 2020	High (low bias and inconsistency, RCT design)
SUGAR-DM-HF 2020	High (low bias and inconsistency, RCT design)
CHIEF-HF 2022	High (low bias and inconsistency, RCT design)
EMPERIAL-REDUCED 2021	High (low bias and inconsistency, RCT design)
DAPA-HF 2019	High (low bias and inconsistency, RCT design)
EMPERIAL-PRESERVED 2021	High (low bias and inconsistency, RCT design)
EMPIRE-HF 2020	High (low bias and inconsistency, RCT design)
DELIVER 2023	High (low bias and inconsistency, RCT design)
EMBRACE-HF 2021	High (low bias and inconsistency, RCT design)
DEFINE-HF 2019	High (low bias and inconsistency, RCT design)
DAPA-VO2 2022	High (low bias and inconsistency, RCT design)
PRESERVED-HF 2021	High (low bias and inconsistency, RCT design)
CANA-HF 2020	Moderate (low bias, moderate risk of inconsistency or impreciseness, RCT design)
Reis et al. 2022	Moderate (low-moderate bias and inconsistency, RCT design)

© 2024 Gao M et al. *JAMA Network Open.*

eTable 2. Conflicts of Interest Table for the 17 Included Studies

Study	Conflicts of Interest Clear?	Study Investigator Initiated?
DEFINE-HF	Yes	Yes
EMPEROR-REDUCED	Yes	No (Boehringer Ingelheim)
DAPA-HF	Yes	No (AstraZeneca)
EMPERIAL-REDUCED	Yes	No (Boehringer Ingelheim)
EMPERIAL-PRESERVED	Yes	No (Boehringer Ingelheim)
CANA-HF	Yes	Yes
SUGAR-DM-HF	Yes	Yes
EMPIRE-HF	Yes	Yes
EMPEROR-PRESERVED	Yes	No (Boehringer Ingelheim)
EMBRACE-HF	Yes	Yes
PRESERVED-HF	Yes	Yes
EMPA-TROPISM	Yes	Yes
CHIEF-HF	Yes	Yes
EMPULSE	Yes	No (Boehringer Ingelheim)
DAPA-VO2	Yes	Yes
Reis et al	Yes	Yes
Deliver	Yes	No (AstraZeneca)

© 2024 Gao M et al. *JAMA Network Open.*

eTable 3. Table of Excluded Studies After Full-Text Review

Article	Lead Author, Year	Reason for Exclusion
Efficacy and Safety of Dapagliflozin in Men and Women With Heart Failure With Reduced Ejection Fraction: A Prespecified Analysis of the Dapagliflozin and Prevention of Adverse Outcomes in Heart Failure Trial	Butt, JH. 2021.	Secondary Analysis
Empagliflozin for Heart Failure With Preserved Left Ventricular Ejection Fraction With and Without Diabetes	Filippatos, G. 2022	Secondary analysis
Effects of sodium-glucose cotransporter-2 inhibitors on nutritional status in heart failure with reduced ejection fraction	Arslan, K. 2022.	No control group with respect to SGLT2i use
Blood Pressure and Dapagliflozin in Heart Failure with Mildly Reduced or Preserved Ejection Fraction: DELIVER	Selvaraj, S. 2023	Secondary Analysis
Cost-effectiveness of immediate initiation of dapagliflozin in patients with a history of heart failure	Miller, R. 2023	Did not analyze outcomes of interest
Renal effects of empagliflozin in patients hospitalized for acute heart failure: from the EMPULSE trial	Voors, A. 2022	Did not analyze outcomes of interest
Endothelin-1, Outcomes in Patients With Heart Failure and Reduced Ejection Fraction, and Effects of Dapagliflozin: Findings From DAPA-HF	Yeoh, S. 2023.	Secondary Analysis
Efficacy and Safety of Dapagliflozin According to Frailty in Patients With Heart Failure: A Prespecified Analysis of the DELIVER Trial	Butt, J. 2022.	Secondary Analysis
Effects of Sodium-Glucose Co-Transporter-2 Inhibition on Pulmonary Arterial Stiffness and Right Ventricular Function in Heart Failure with Reduced Ejection Fraction	Camci, S. 2022	No control group with respect to SGLT2i use
Efficacy of empagliflozin in heart failure with preserved versus mid-range ejection fraction: a pre-specified analysis of EMPEROR-Preserved	Anker, S. 2022	Secondary Analysis
Impact of Empagliflozin in Heart Failure With Reduced Ejection Fraction in Patients With Ischemic Versus Nonischemic Cause	Khan, M. 2023	Secondary Analysis
Early benefit with empagliflozin in heart failure with preserved ejection fraction: insights from the EMPEROR-Preserved trial	Butler, J. 2022	Commentary
ADDition of DAPAgliflozin, Sodium-Glucose Cotransporter-2 Inhibitor to Angiotensin Receptor Blocker-Neprilysin Inhibitors Non-Responders in Patient with Refractory Heart Failure with Reduced Ejection Fraction (ADD DAPA trial)	Jariwala, P. 2022	Did not analyze outcomes of interest

© 2024 Gao M et al. *JAMA Network Open.*

Efficacy of Dapagliflozin on Renal Function and Outcomes in Patients With Heart Failure With Reduced Ejection Fraction: Results of DAPA-HF	Jhund, P. 2021	Did not analyze outcomes of interest and secondary analysis
Dapagliflozin for heart failure according to body mass index: the DELIVER trial	Adamson, C. 2022	Secondary Analysis
[Impact of empagliflozin on peak oxygen uptake in HFmrEF patients: a randomized controlled trial]	Wu, W. 2022	Unable to access full article beyond abstract
Dapagliflozin and atrial fibrillation in heart failure with reduced ejection fraction: insights from DAPA-HF	Butt, J. 2022	Secondary Analysis
Dapagliflozin in patients with heart failure with mildly reduced and preserved ejection fraction treated with a mineralocorticoid receptor antagonist or sacubitril/valsartan	Yang, M. 2022	Secondary Analysis
Atrial Fibrillation and Dapagliflozin Efficacy in Patients With Preserved or Mildly Reduced Ejection Fraction	Butt, J. 2022	Secondary Analysis
Dapagliflozin in HFrEF Patients Treated With Mineralocorticoid Receptor Antagonists: An Analysis of DAPA-HF	Shen, L. 2021	Secondary Analysis
IGFBP-7 and Outcomes in Heart Failure With Reduced Ejection Fraction: Findings From DAPA-HF	Adamson, C. 2023	Secondary Analysis
Effects of empagliflozin on cardiorespiratory fitness and significant interaction of loop diuretics	Carbone, S. 2018	No control group with respect to SGLT2i use
Empagliflozin Improves Outcomes in Patients With Heart Failure and Preserved Ejection Fraction Irrespective of Age	Bohm, M. 2022	Secondary Analysis
Effects of dapagliflozin in heart failure with reduced ejection fraction and chronic obstructive pulmonary disease: an analysis of DAPA-HF	Dewan, P. 2021	Secondary Analysis
Efficacy and safety of sodium-glucose co-transporter 2 inhibition according to left ventricular ejection fraction in DAPA-HF	Dewan, P. 2020	Secondary Analysis
Association between serum insulin levels and heart failure-related parameters in patients with type 2 diabetes and heart failure treated with canagliflozin: a post-hoc analysis of the randomized CANDLE trial	Tanaka, A. 2022	Did not analyze outcomes of interest and secondary analysis
Safety and Efficacy of the Combination of Sacubitril/Valsartan and SGLT2i in HFrEF Patients (SECSI Registry)	Jiménez-Blanco Bravo, M 2021.	Did not analyze outcomes of interest
Dapagliflozin in Patients Recently Hospitalized With Heart Failure and Mildly Reduced or Preserved Ejection Fraction	Cunningham, J. 2022	Did not analyze outcomes of interest
Empagliflozin improves quality of life in nondiabetic HFrEF patients. Sub-analysis of the EMPATROPISM trial	Requena-Ibáñez, J. A. 2022	Secondary Analysis
Efficacy of Dapagliflozin in Black Versus White Patients With Heart Failure and Reduced Ejection Fraction	Doherty, K. 2022	Secondary Analysis
Association of Dapagliflozin Use With Clinical Outcomes and the Introduction of	Butt, J. 2023	Secondary Analysis

© 2024 Gao M et al. *JAMA Network Open.*

Uric Acid-Lowering Therapy and Colchicine in Patients With Heart Failure With and Without Gout: A Patient-Level Pooled Meta-analysis of DAPA-HF and DELIVER		
Different Doses of Empagliflozin in Patients with Heart Failure with Reduced Ejection Fraction	Hao, Z. 2022	Did not analyze outcomes of interest.
Effects of 6 weeks of treatment with dapagliflozin, a sodium-glucose co-transporter-2 inhibitor, on myocardial function and metabolism in patients with type 2 diabetes: A randomized, placebo-controlled, exploratory study	Oldgren, J. 2021	Did not analyze outcomes of interest.
Effect of canagliflozin on N-terminal pro-brain natriuretic peptide in patients with type 2 diabetes and chronic heart failure according to baseline use of glucose-lowering agents	Tanaka, A. 2021	Secondary analysis and did not analyze outcomes of interest
Initiation of New Glucose-Lowering Therapies May Act to Reduce Physical Activity Levels: Pooled Analysis From Three Randomized Trials	Yates, T. 2022	Secondary analysis
SGLT2 inhibitors in patients with heart failure with reduced ejection fraction: a meta-analysis of the EMPEROR-Reduced and DAPA-HF trials	Zannad, F. 2020	Secondary analysis
Empagliflozin in Black Versus White Patients With Heart Failure: Analysis of EMPEROR-Pooled	Verma, S. 2023	Secondary analysis
Efficacy and Safety of Dapagliflozin According to Frailty in Heart Failure With Reduced Ejection Fraction : A Post Hoc Analysis of the DAPA-HF Trial	Butt, J. 2022	Secondary analysis
Early effects of empagliflozin on exercise tolerance in patients with heart failure: A pilot study	Nunez, J. 2018	No control or placebo group
Efficacy and safety of dapagliflozin according to aetiology in heart failure with reduced ejection fraction: insights from the DAPA-HF trial	Butt, J. 2021	Secondary analysis
Impact of empagliflozin on decongestion in acute heart failure: the EMPULSE trial	Biergus, J. 2023	Secondary analysis
Effect of Dapagliflozin on Cardiac Function and Metabolic and Hormonal Responses to Exercise	Herring, R. 2023	Did not analyze outcomes of interest
Patient Characteristics, Outcomes, and Effects of Dapagliflozin According to the Duration of Heart Failure: A Prespecified Analysis of the DELIVER Trial	Kondo, T. 2023	Secondary analysis
Influence of NT-proBNP on Efficacy of Dapagliflozin in Heart Failure With Mildly Reduced or Preserved Ejection Fraction	Myrhe, P. 2022	Secondary analysis
Weight change and clinical outcomes in heart failure with reduced ejection fraction: insights from EMPEROR-Reduced	Anker, S. 2023	Secondary analysis
Efficacy of dapagliflozin in heart failure with reduced ejection fraction according to body mass index	Adamson, C. 2023	Did not analyze outcomes of interest

© 2024 Gao M et al. *JAMA Network Open.*

Safety and efficacy of dapagliflozin in patients with focal segmental glomerulosclerosis: a prespecified analysis of the dapagliflozin and prevention of adverse outcomes in chronic kidney disease (DAPA-CKD) trial	Wheeler, D. 2022	Did not analyze outcomes of interest
Effect of dapagliflozin on anaemia in DAPA-HF	Docherty, K. 2021	Secondary analysis
Efficacy of SGLT2 Inhibitors in Patients With Diabetes and Nonobstructive Hypertrophic Cardiomyopathy	Subramarian, M. 2023	Focused on patients with HOCM, which was ultimately not within the scope of our meta-analysis
Effect of Empagliflozin on Cardiovascular and Renal Outcomes in Patients With Heart Failure by Baseline Diabetes Status: Results From the EMPEROR-Reduced Trial	Anker, S. 2021	Secondary analysis
Iron Deficiency in Heart Failure and Effect of Dapagliflozin: Findings From DAPA-HF	Docherty, K. 2021	Secondary analysis
SGLT2 Inhibition Does Not Affect Myocardial Fatty Acid Oxidation or Uptake, but Reduces Myocardial Glucose Uptake and Blood Flow in Individuals With Type 2 Diabetes: A Randomized Double-Blind, Placebo-Controlled Crossover Trial	Lauritsen, K. 2021	Did not analyze outcomes of interest
Empagliflozin Improves Cognitive Impairment in Frail Older Adults With Type 2 Diabetes and Heart Failure With Preserved Ejection Fraction	Mone, P. 2022	Did not analyze outcomes of interest
Dapagliflozin and Diuretic Use in Patients With Heart Failure and Reduced Ejection Fraction in DAPA-HF	Jackson, A. 2020	Secondary analysis
Effects of Empagliflozin in Women and Men With Heart Failure and Preserved Ejection Fraction	Butler, J. 2022	Secondary analysis
Interplay of Mineralocorticoid Receptor Antagonists and Empagliflozin in Heart Failure: EMPEROR-Reduced	Ferreira, J. 2021	Secondary analysis
Sex Differences in Characteristics, Outcomes, and Treatment Response With Dapagliflozin Across the Range of Ejection Fraction in Patients With Heart Failure: Insights From DAPA-HF and DELIVER	Wang, X. 2023	Secondary analysis
Liver tests and outcomes in heart failure with reduced ejection fraction: findings from DAPA-HF	Adamson, C. 2022	Secondary analysis
Effects of ipragliflozin on left ventricular diastolic function in patients with type 2 diabetes and heart failure with preserved ejection fraction: The EXCEED randomized controlled multicenter study	Akasaka, H. 2022	Did not analyze outcomes of interest
Quality of life in EMPEROR-Reduced: emphasizing what is important to patients while identifying strategies to support more patient-centred care	Spertus, J. 2022	Editorial commentary
Empagliflozin in heart failure with preserved and mildly reduced ejection fraction: prognostic benefit confirmed with different endpoint definitions	Bayis-Genis A. 2022	Editorial Commentary

Effect of dapagliflozin according to baseline systolic blood pressure in the Dapagliflozin and Prevention of Adverse Outcomes in Heart Failure trial (DAPA-HF)	Serenelli, M. 2020	Secondary analysis
Dapagliflozin and New York Heart Association functional class in heart failure with mildly reduced or preserved ejection fraction: the DELIVER trial	Ostrominski, J. 2022	Secondary analysis
Efficacy and Safety of Dapagliflozin in Heart Failure With Reduced Ejection Fraction According to N-Terminal Pro-B-Type Natriuretic Peptide: Insights From the DAPA-HF Trial	Butt, J. 2021	Secondary analysis
Empagliflozin in patients hospitalized for acute heart failure	Ponikowski, P. 2022	Editorial commentary
Influence of neprilysin inhibition on the efficacy and safety of empagliflozin in patients with chronic heart failure and a reduced ejection fraction: the EMPEROR-Reduced trial	Packer, M.	Secondary analysis
Dapagliflozin in Black and White Patients With Heart Failure Across the Ejection Fraction Spectrum	Butt, J. 2023	Secondary analysis
Patient Characteristics, Clinical Outcomes, and Effect of Dapagliflozin in Relation to Duration of Heart Failure: Is It Ever Too Late to Start a New Therapy?	Yeoh, S. 2020	Secondary analysis
Short-term Changes in Hemoglobin and Changes in Functional Status, Quality of Life and Natriuretic Peptides After Initiation of Dapagliflozin in Heart Failure With Reduced Ejection Fraction	Lorenzo, M. 2023	Secondary analysis
Recency of Heart Failure Hospitalization, Outcomes, and the Effect of Empagliflozin: An EMPEROR-Pooled Analysis	Ferreira, J. 2023	Secondary analysis
Relationship of Dapagliflozin With Serum Sodium: Findings From the DAPA-HF Trial	Yeoh, S. 2022	Secondary analysis
Influence of Sodium Glucose Cotransporter 2 Inhibition on Physiological Adaptation to Endurance Exercise Training	Newman, A. 2019	Explicitly excluded any patients with cardiac or cardiovascular pathology
Serial Assessment of High-Sensitivity Cardiac Troponin and the Effect of Dapagliflozin in Patients With Heart Failure With Reduced Ejection Fraction: An Analysis of the DAPA-HF Trial	Berg, D. 2022	Secondary analysis
Effect of Dapagliflozin in Patients With HFrEF Treated With Sacubitril/Valsartan: The DAPA-HF Trial	Solomon, S. 2020	Secondary analysis
Impact of anaemia and the effect of empagliflozin in heart failure with reduced ejection fraction: findings from EMPEROR-Reduced	Ferreira, J. 2022	Secondary analysis
Effect of Dapagliflozin on Worsening Heart Failure and Cardiovascular Death in Patients With Heart Failure With and Without Diabetes	Petrie, M. 2020	Secondary analysis

© 2024 Gao M et al. *JAMA Network Open.*

Association Between Change in Ambulatory Hemodynamic Pressures and Symptoms of Heart Failure	Nassif, M. 2021	Did not include outcomes of interest or patients treated with SGLT2 as separate treatment group
Concentration-dependent clinical and prognostic importance of high-sensitivity cardiac troponin T in heart failure and a reduced ejection fraction and the influence of empagliflozin: the EMPEROR-Reduced trial	Packer, M. 2021	Secondary analysis
Effect of empagliflozin in patients with heart failure across the spectrum of left ventricular ejection fraction	Butler, J. 2022	Secondary analysis
Mineralocorticoid Receptor Antagonists and Empagliflozin in Patients With Heart Failure and Preserved Ejection Fraction	Ferreira, J. 2022	Secondary analysis
Effects of the dual sodium-glucose linked transporter inhibitor, licogliflozin vs placebo or empagliflozin in patients with type 2 diabetes and heart failure	De Boer, R. 2022	Did not analyze outcomes of interest
Regional and ethnic influences on the response to empagliflozin in patients with heart failure and a reduced ejection fraction: the EMPEROR-Reduced trial	Lam, C. 2021.	Secondary analysis
Empagliflozin improves cardiorespiratory fitness in type 2 diabetes: translational implications	Kumar, N. 2018	Could not analyze results of control/placebo group
Effects of Dapagliflozin According to the Heart Failure Collaboratory Medical Therapy Score: Insights From DAPA-HF	Butt, J. 2022	Secondary analysis
Efficacy and Safety of Dapagliflozin in Heart Failure With Reduced Ejection Fraction According to Age: Insights From DAPA-HF	Martinez, F. 2020	Secondary analysis
Efficacy of Empagliflozin in Patients With Heart Failure Across Kidney Risk Categories	Butler, J. 2023	Secondary analysis
Prognostic Implications of N-Terminal Pro-B-Type Natriuretic Peptide and High-Sensitivity Cardiac Troponin T in EMPEROR-Preserved	Januzzi, J. 2022	Secondary analysis
Metabolomic Profiling of the Effects of Dapagliflozin in Heart Failure With Reduced Ejection Fraction: DEFINE-HF	Selvaraj, S. 2022	Secondary analysis
Effects of empagliflozin on cardiovascular and renal outcomes in heart failure with reduced ejection fraction according to age: a secondary analysis of EMPEROR-Reduced	Filippatos, G. 2022	Secondary analysis

© 2024 Gao M et al. *JAMA Network Open.*

eFigure 2. Subgroup Analyses: 6-MWD

A. Subgroup Analysis Stratified by SGLT2i Used in the Trial

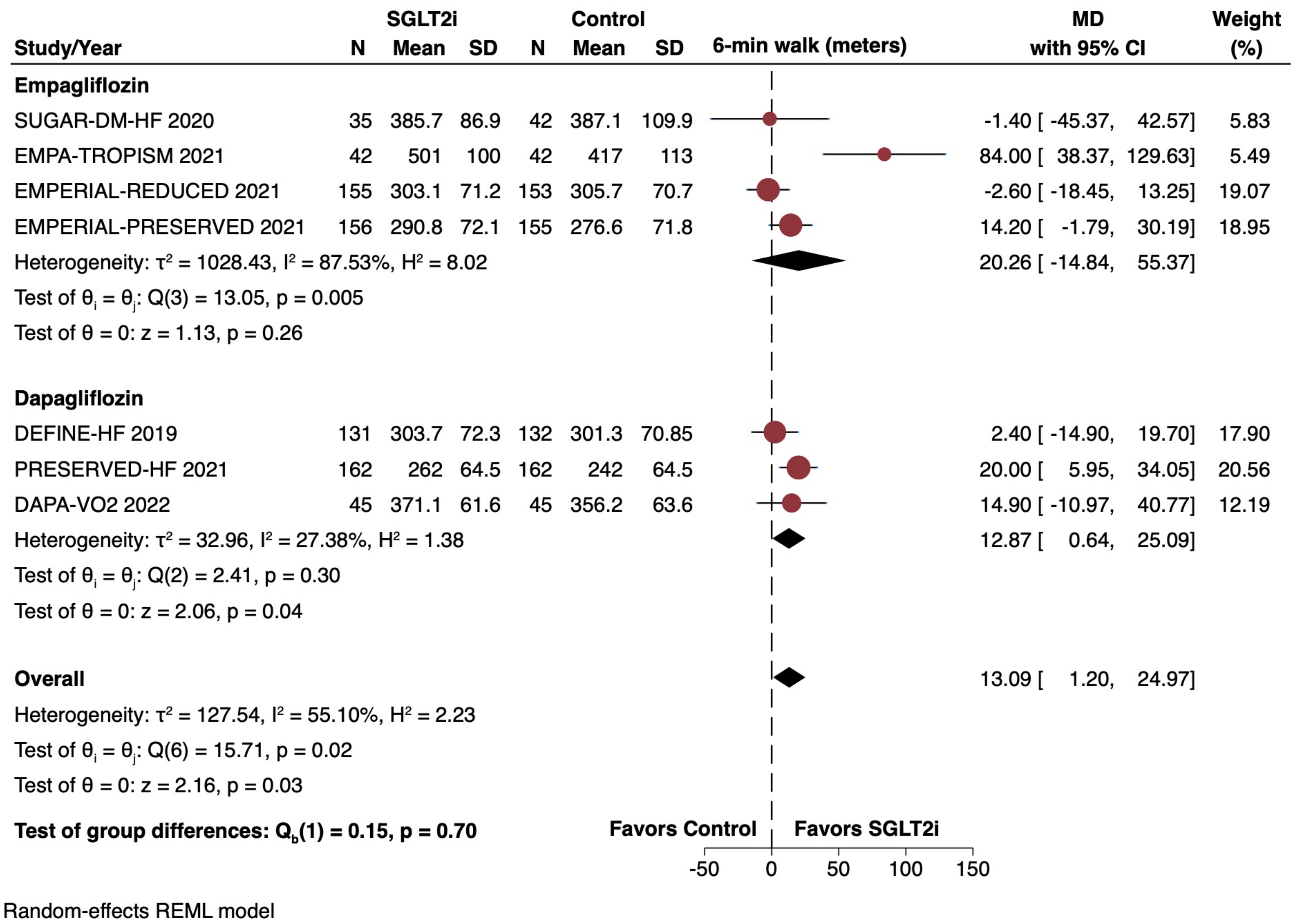

© 2024 Gao M et al. *JAMA Network Open.*

B. Subgroup Analysis Stratified by Ejection Fraction

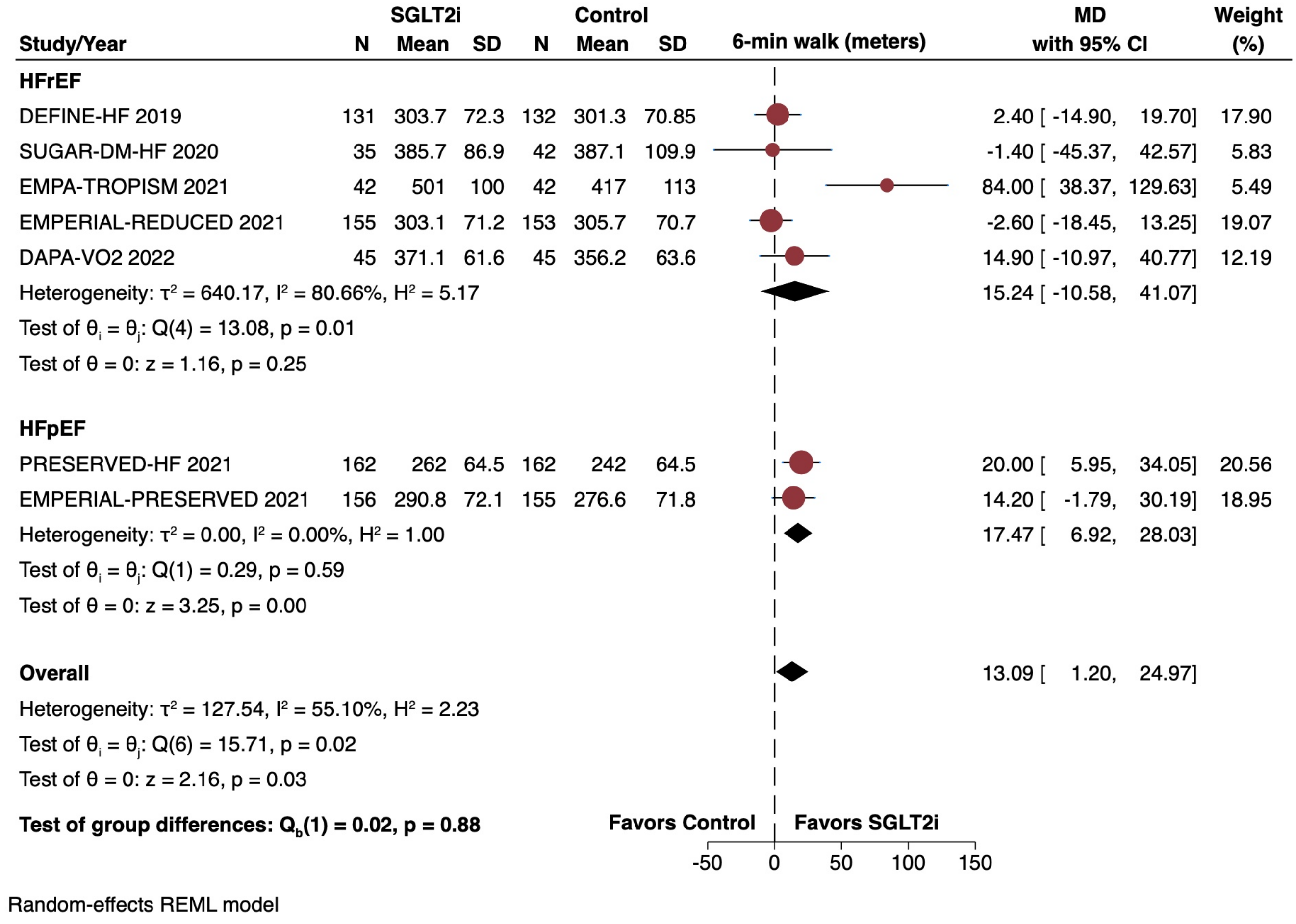

© 2024 Gao M et al. *JAMA Network Open.*

C. Subgroup Analysis by Follow-Up Duration

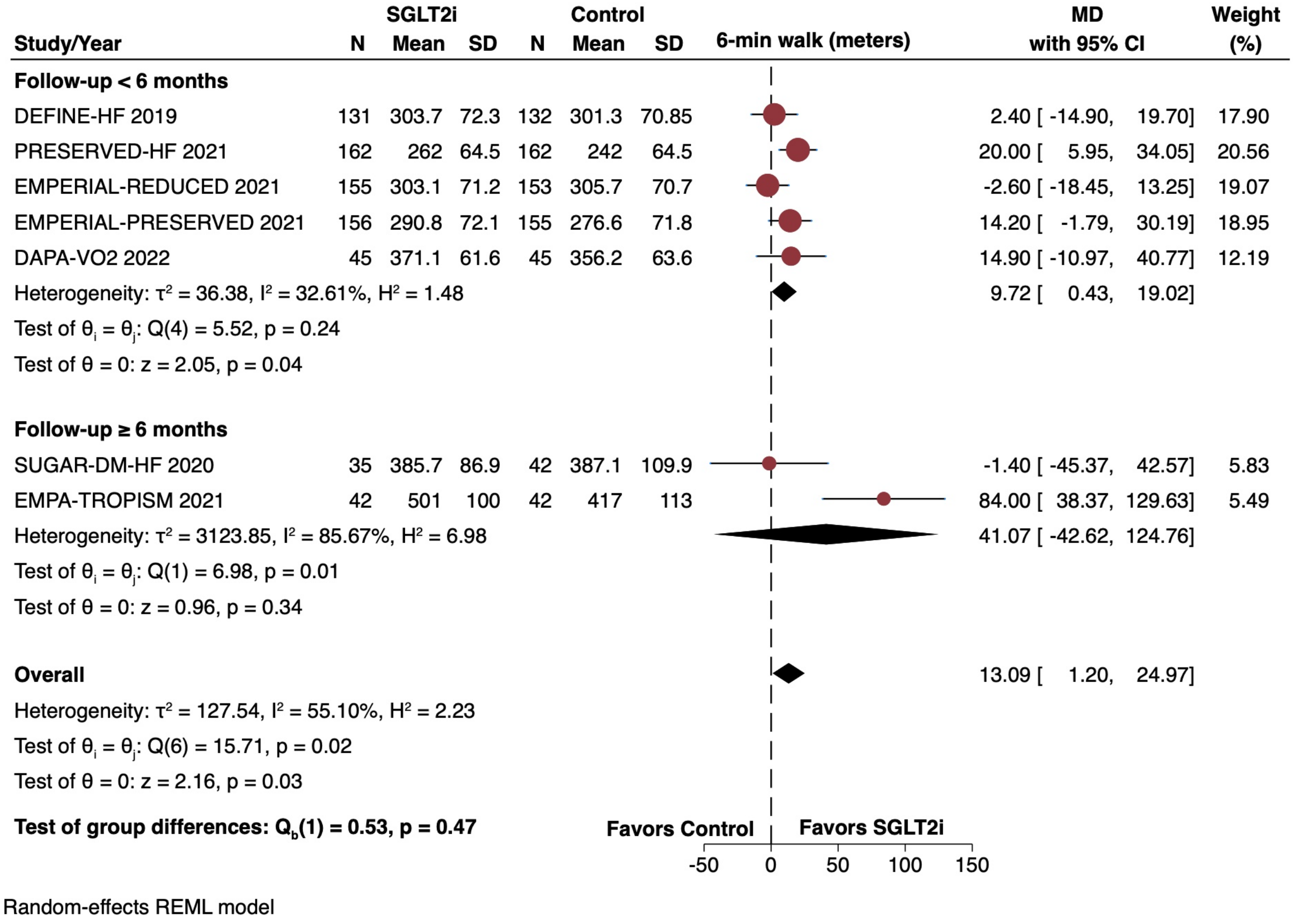

© 2024 Gao M et al. *JAMA Network Open.*

eFigure 3. Bubble Plots by Proportion of Females and Diabetic Patients Included in Trials Reporting 6-MWD

A. Bubble plot for 6-MWD and Proportion of Females Included in Each Trial

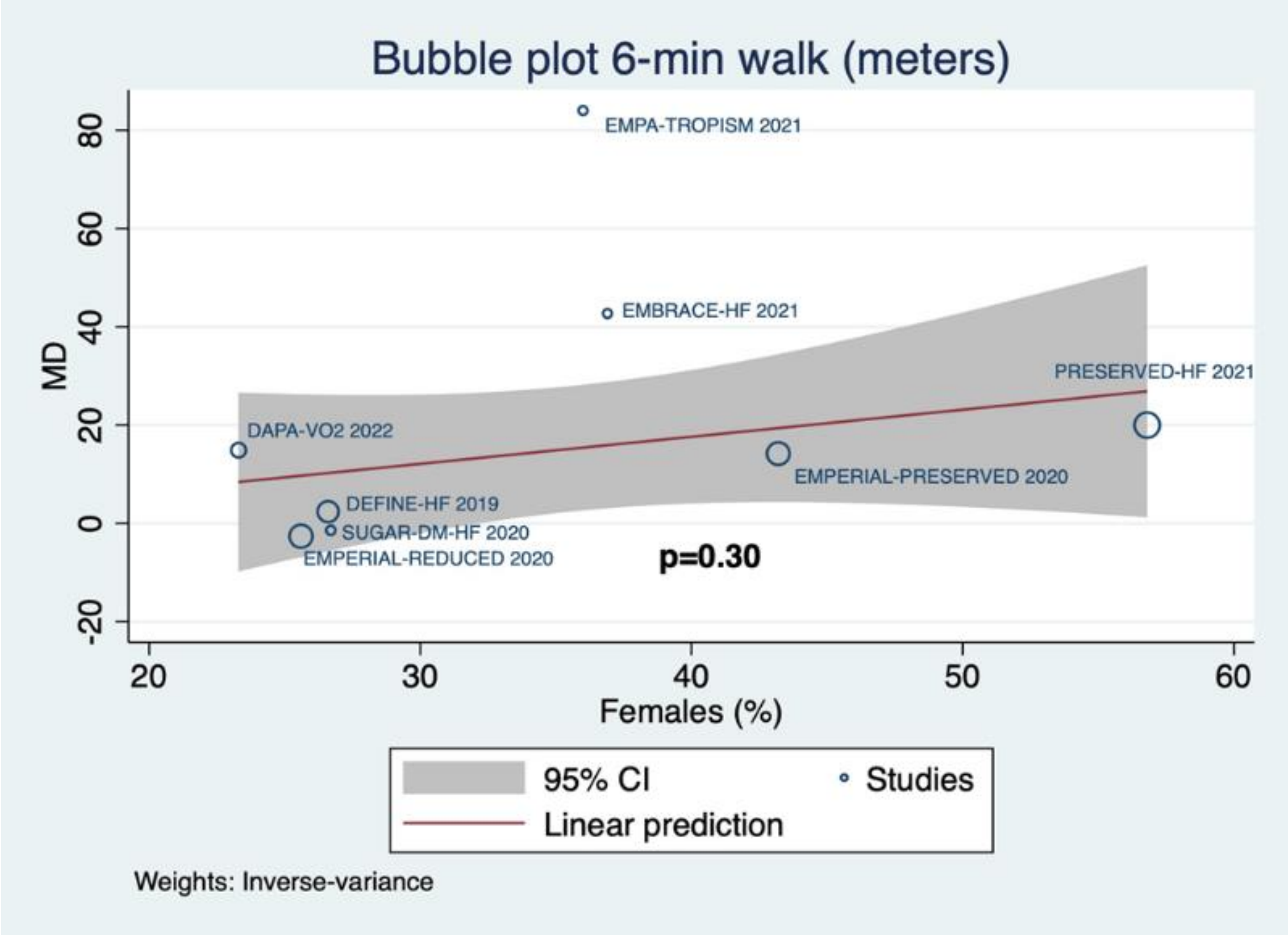

© 2024 Gao M et al. *JAMA Network Open.*

B. Bubble Plot 6-MWD and Proportion of Type 2 Diabetic Patients Included in Each Trial

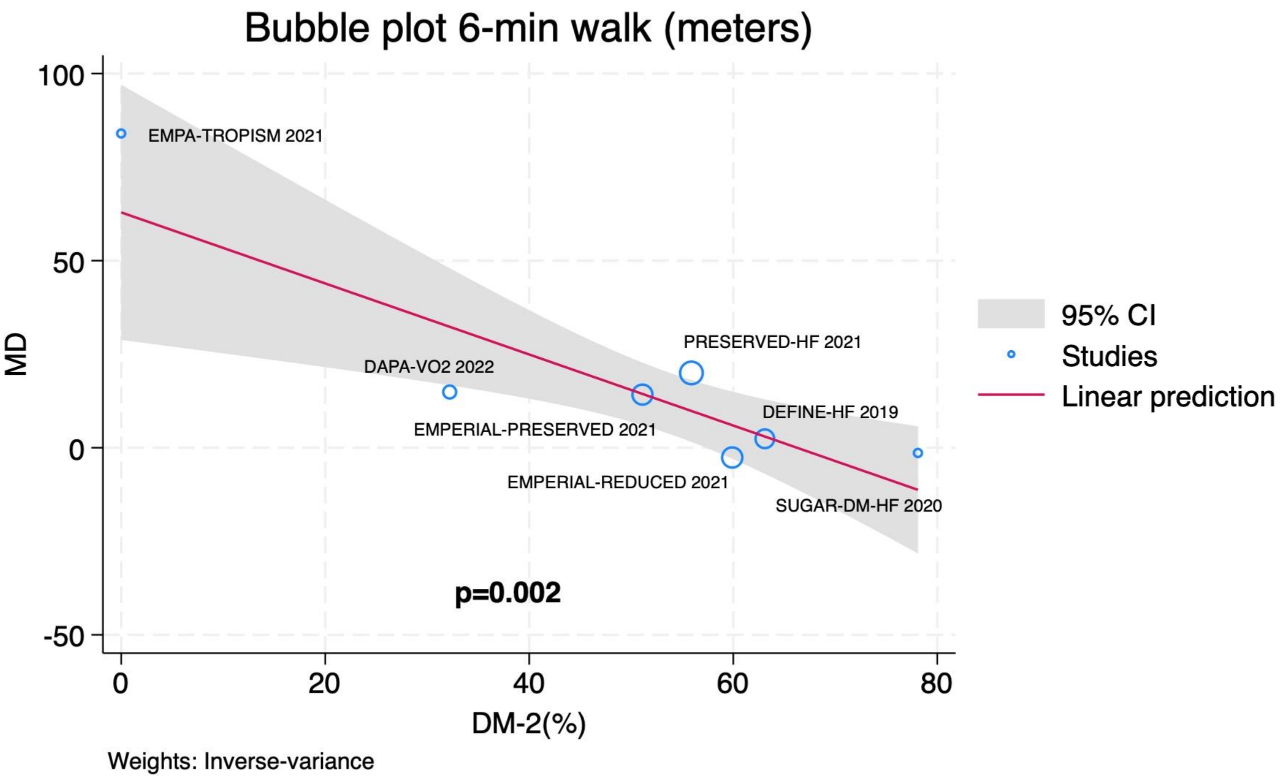

© 2024 Gao M et al. *JAMA Network Open.*

eTable 4. Studies That Reported Means and 95% Confidence Intervals for the 6-MWD at the End of Follow-Up Used to Derive Standard Deviations

Study	Patients in SGLT2i arm	Mean and 95% Confidence Interval for SGLT2i	Patients in Control arm	Mean and 95% Confidence Interval for SGLT2i
DEFINE-HF 2019[20]	131	303.7 (291.2,316.7)	132	301.3 (289.1, 313.9)
PRESERVED-HF 2021[27]	162	262 (252, 272	162	242 (232, 252)
EMPERIAL REDUCED 2021[24]	155	303.1 (291.8, 314.3)	153	305.7 (294.4, 316.9)
EMPERIAL PRESERVED 2021[24]	156	290.8 (279.4, 302.3)	155	276.6 (265.2, 288.0)
DAPA-VO2 2022[31]	45	371.1 (352.6–389.6)	45	356.2 (337.1–375.3)

The standard deviation was calculated using the number of patients, mean difference, and 95% confidence interval through the calculator provided in the Revman 5.3. Only studies with symmetrical confidence intervals around the mean were included in the pooled analysis, in accordance with the Cochrane handbook chapter on obtaining standard deviations from confidence intervals from group means.
https://handbook51.cochrane.org/chapter_7/7_7_3_2_obtaining_standard_deviations_from_standard_errors_and.html.

© 2024 Gao M et al. *JAMA Network Open.*

eFigure 4. Subgroup Analyses: KCCQ-TSS

A. Subgroup Analysis Stratified by SGLT2i Used in the Trial

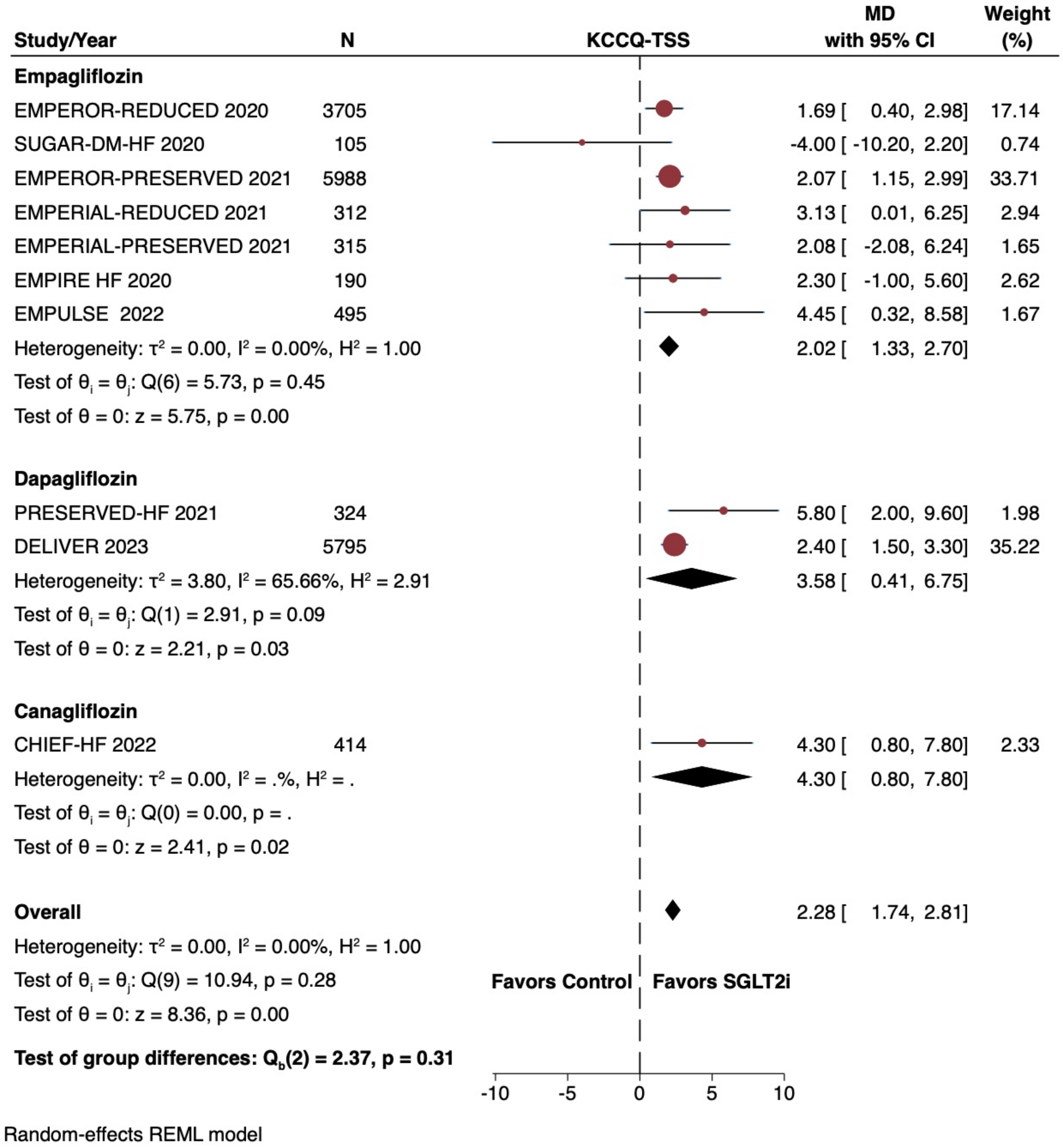

© 2024 Gao M et al. *JAMA Network Open.*

B. Subgroup Analysis Stratified by Ejection Fraction

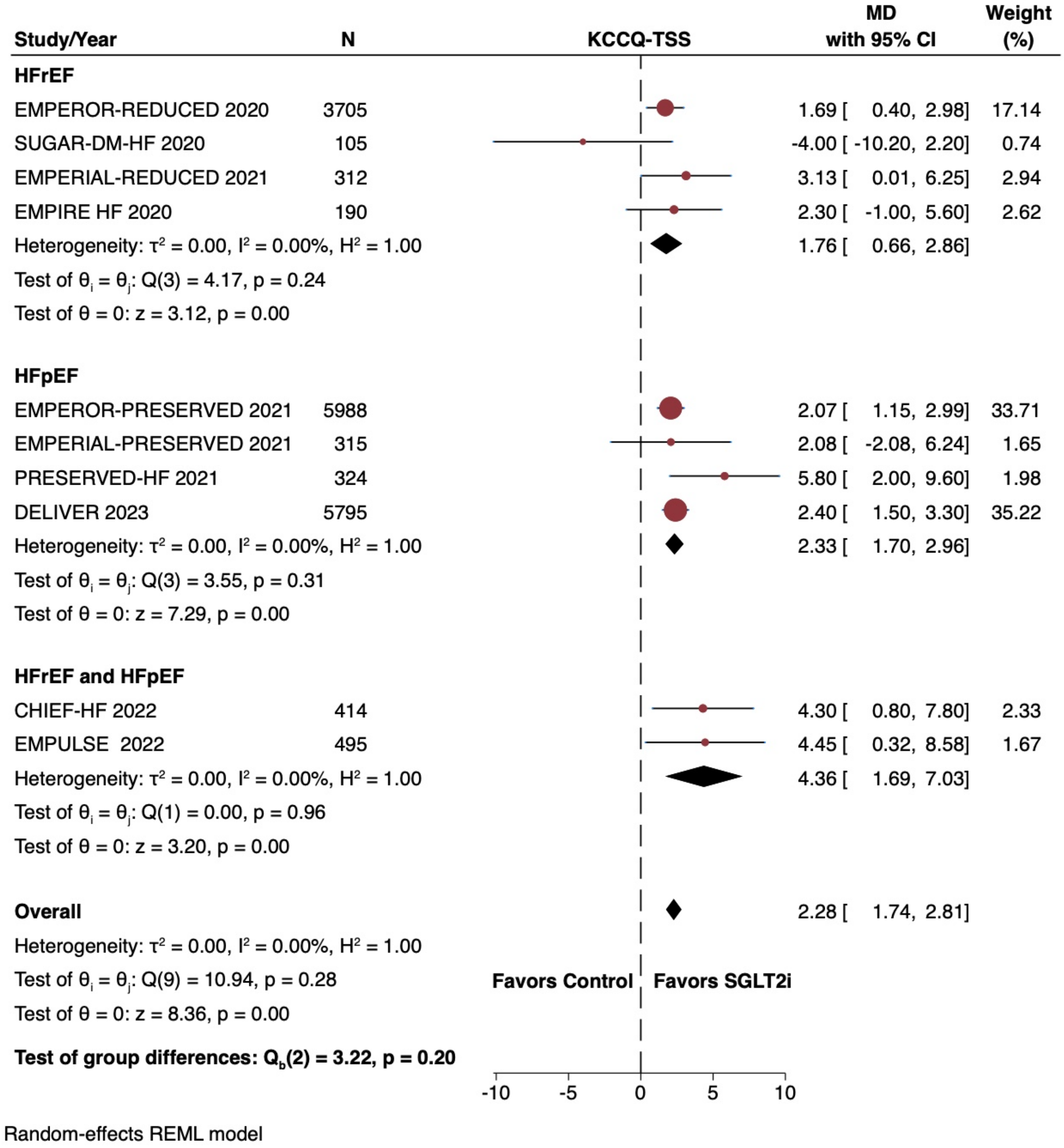

© 2024 Gao M et al. *JAMA Network Open.*

C. Subgroup Analysis by Follow-Up Duration

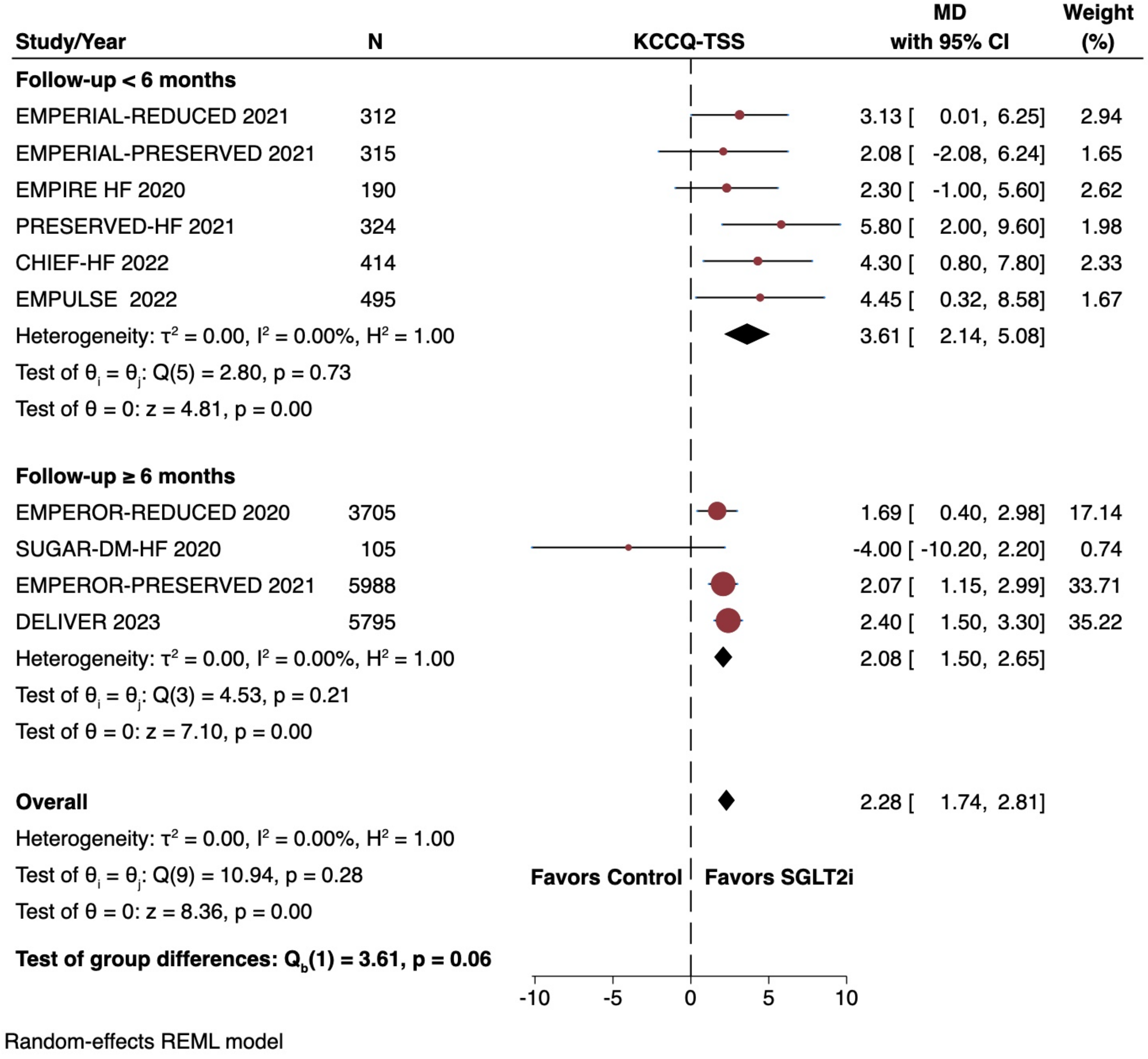

© 2024 Gao M et al. *JAMA Network Open.*

eFigure 5. Subgroup Analysis: KCCQ-OS
A. Subgroup Analysis Stratified by SGLT2i Used in the Trial

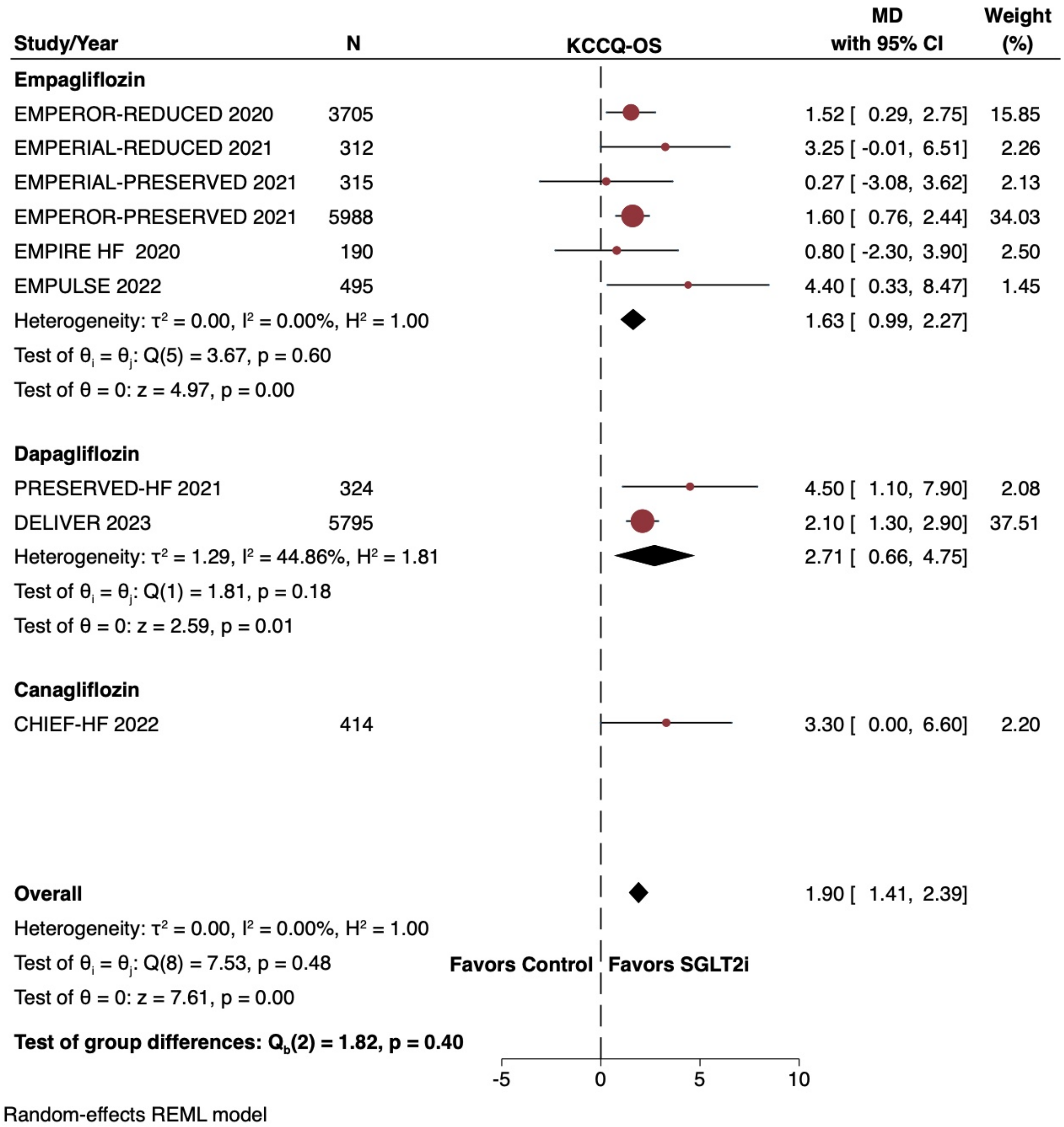

© 2024 Gao M et al. *JAMA Network Open.*

B. Subgroup Analysis Stratified by Ejection Fraction

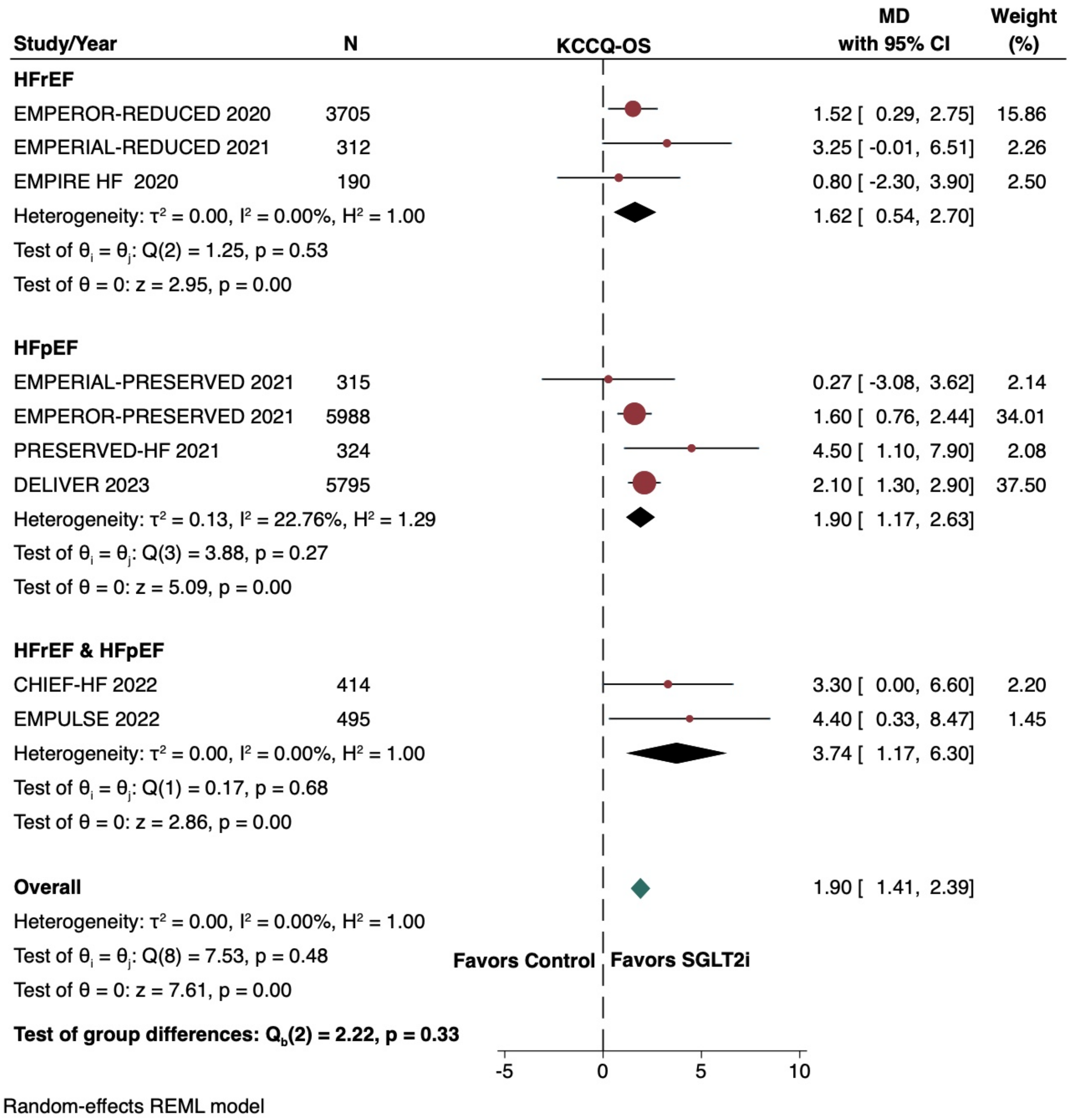

© 2024 Gao M et al. *JAMA Network Open.*

C. Subgroup Analysis by Follow-Up Duration

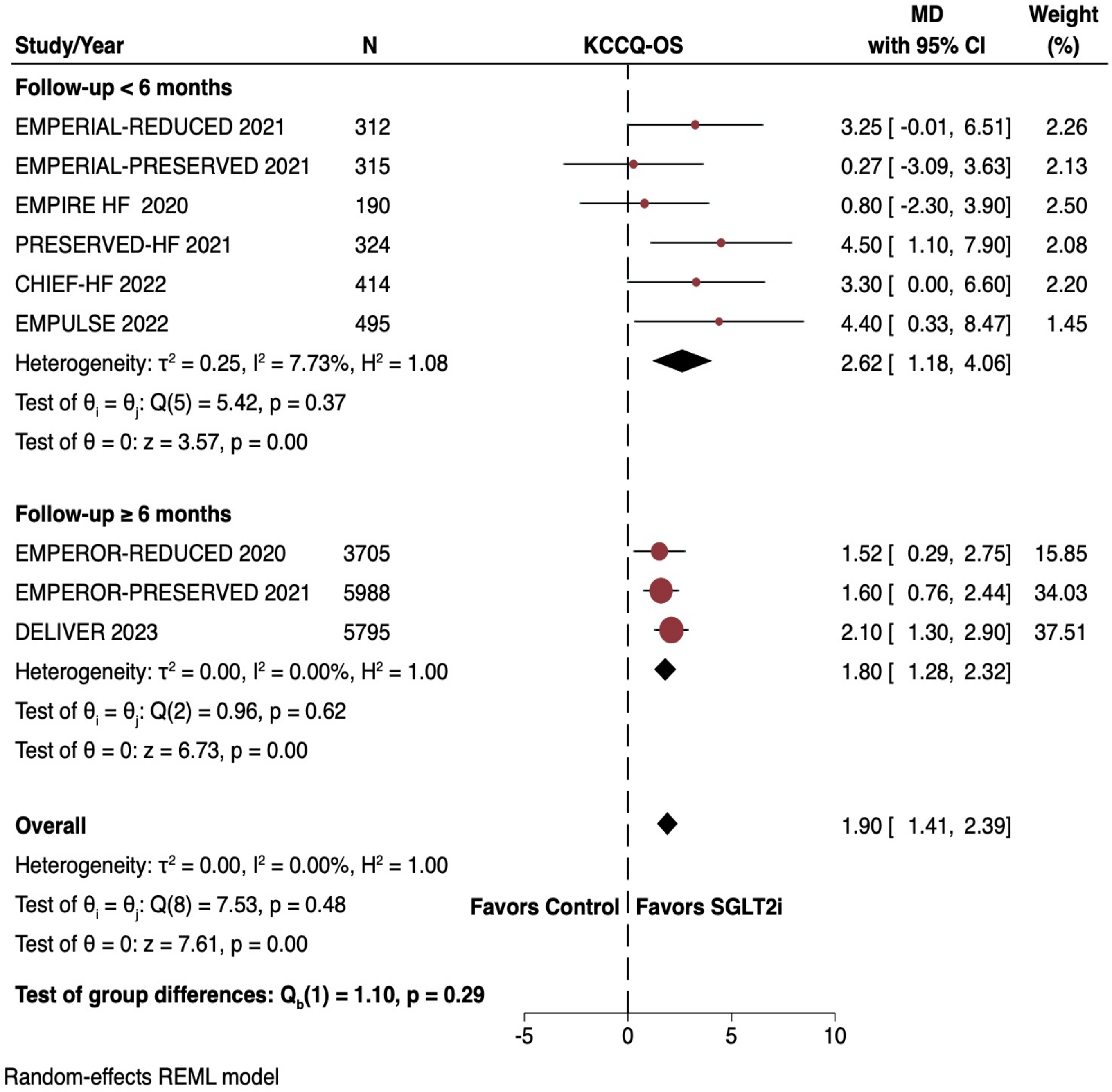

© 2024 Gao M et al. *JAMA Network Open.*

eFigure 6. Subgroup Analyses: KCCQ-CSS

A. Subgroup Analysis Stratified by SGLT2i Used in the Trial

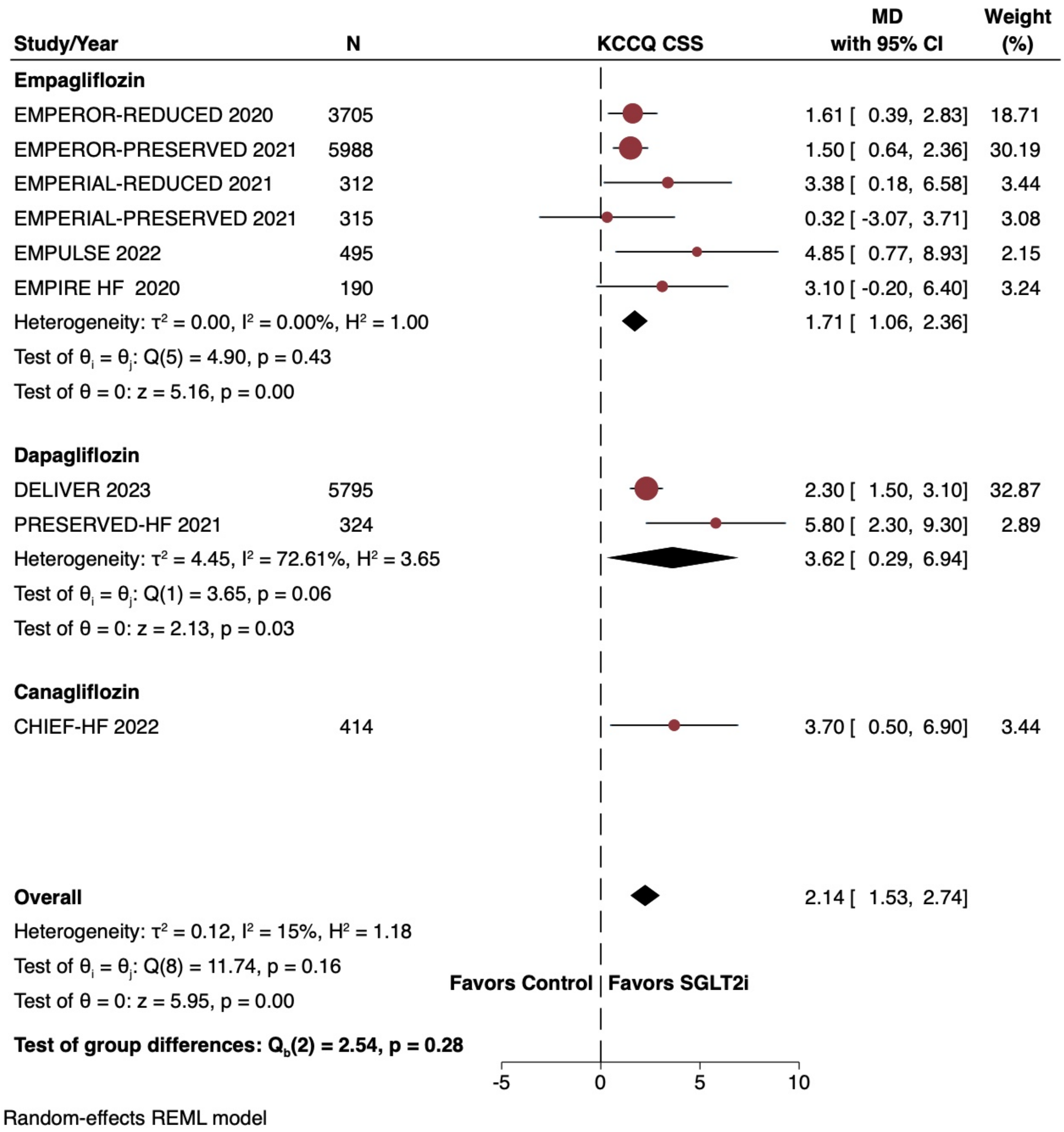

© 2024 Gao M et al. *JAMA Network Open.*

B. Subgroup Analysis Stratified by Ejection Fraction

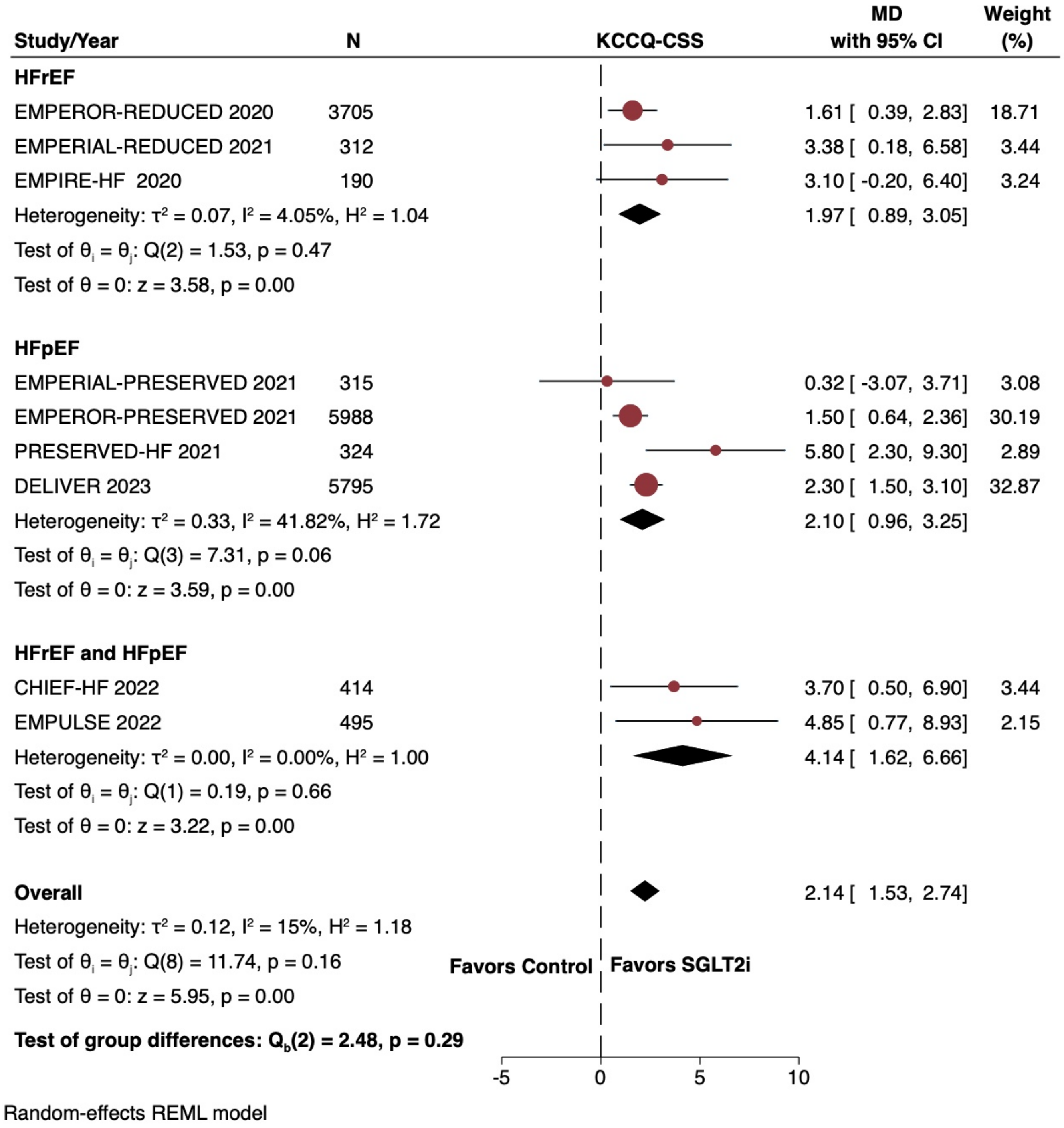

© 2024 Gao M et al. *JAMA Network Open.*

C. Subgroup Analysis by Follow-Up Duration

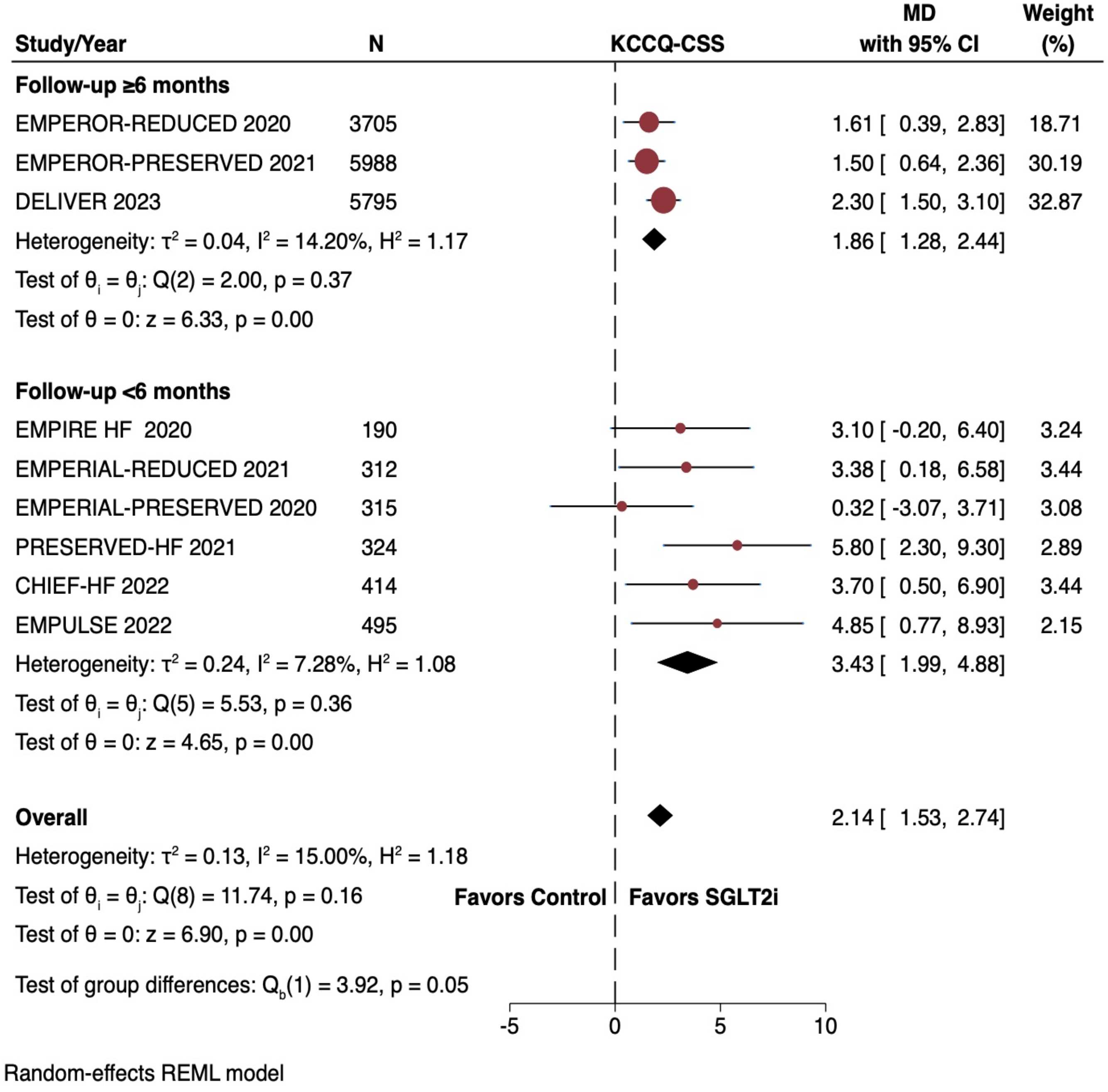

© 2024 Gao M et al. *JAMA Network Open.*

eFigure 7. Pooled Results of Improvement in KCCQ Scores

A. Improvement in KCCQ- OS Scores by ≥ 5 Points

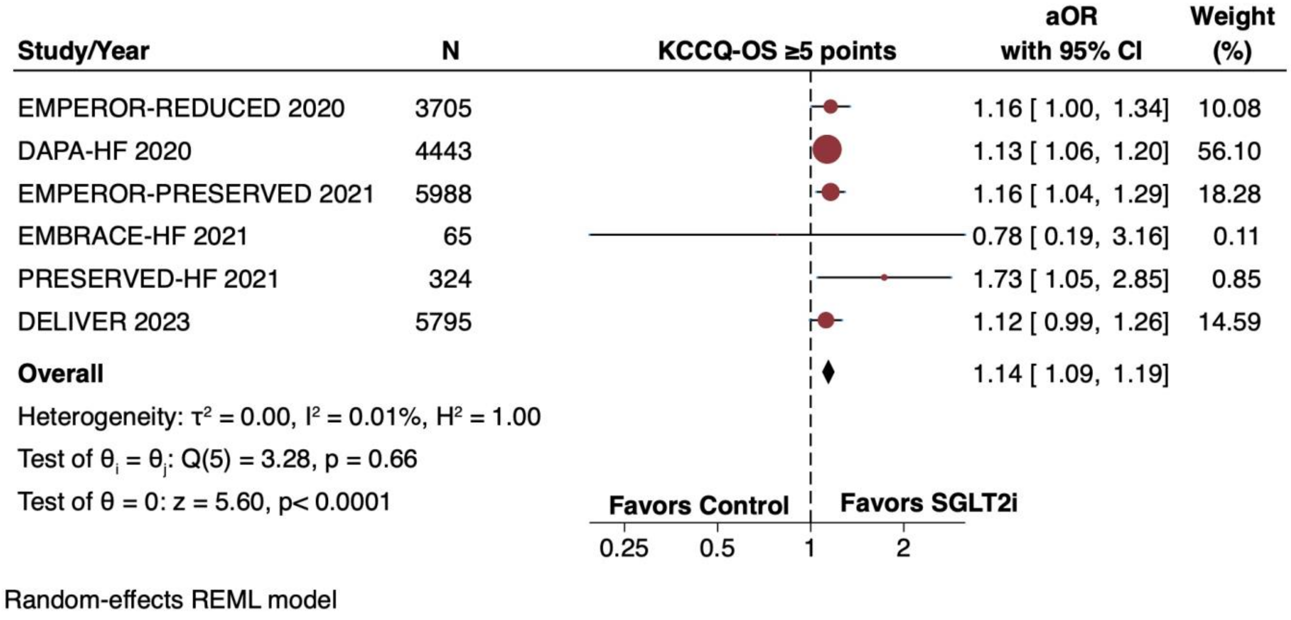

B. Improvement in KCCQ- OS Scores by ≥ 10 Points

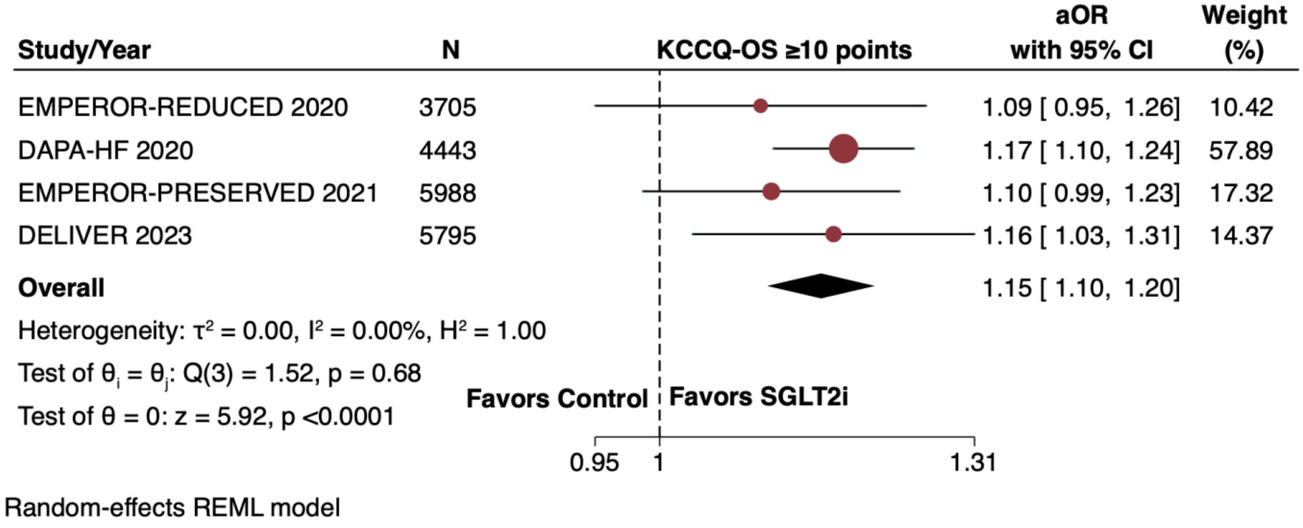

© 2024 Gao M et al. *JAMA Network Open.*

C. Improvement in KCCQ- OS Scores by ≥ 15 Points

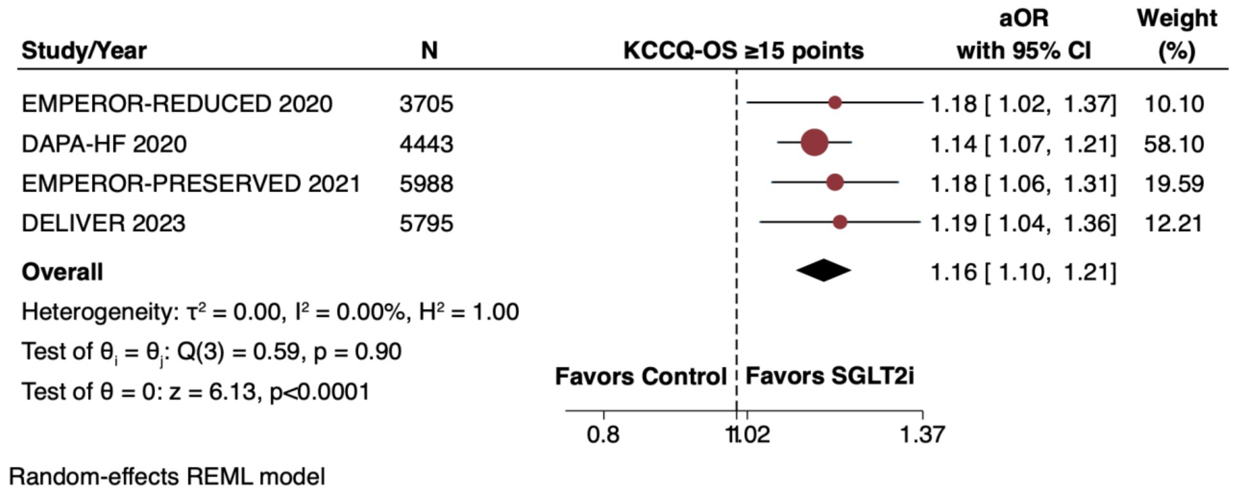

D. Improvement in KCCQ-TSS Scores by ≥ 5 Points

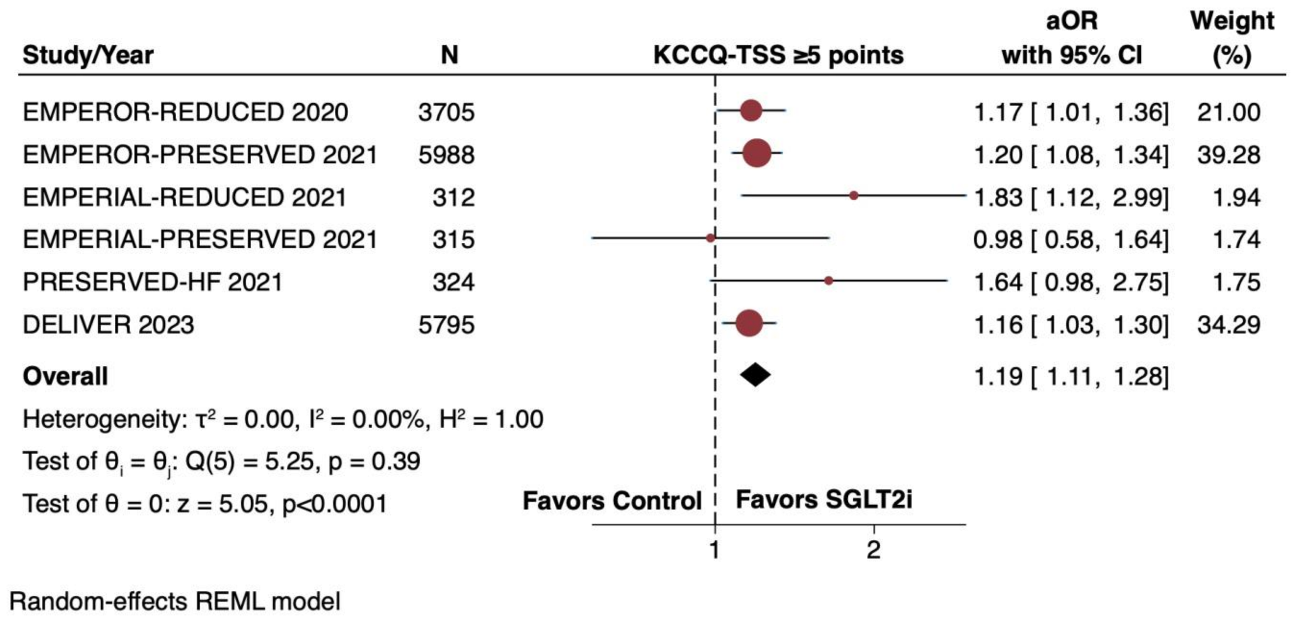

© 2024 Gao M et al. *JAMA Network Open.*

E. Improvement in KCCQ- TSS Scores by ≥ 10 Points

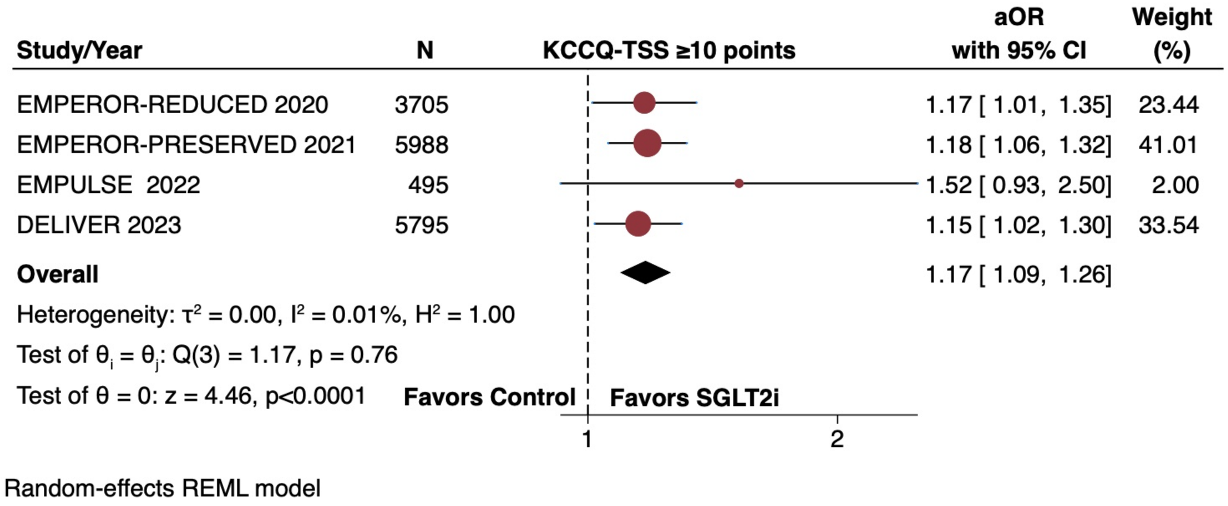

F. Improvement in KCCQ- TSS Scores by ≥ 15 Points

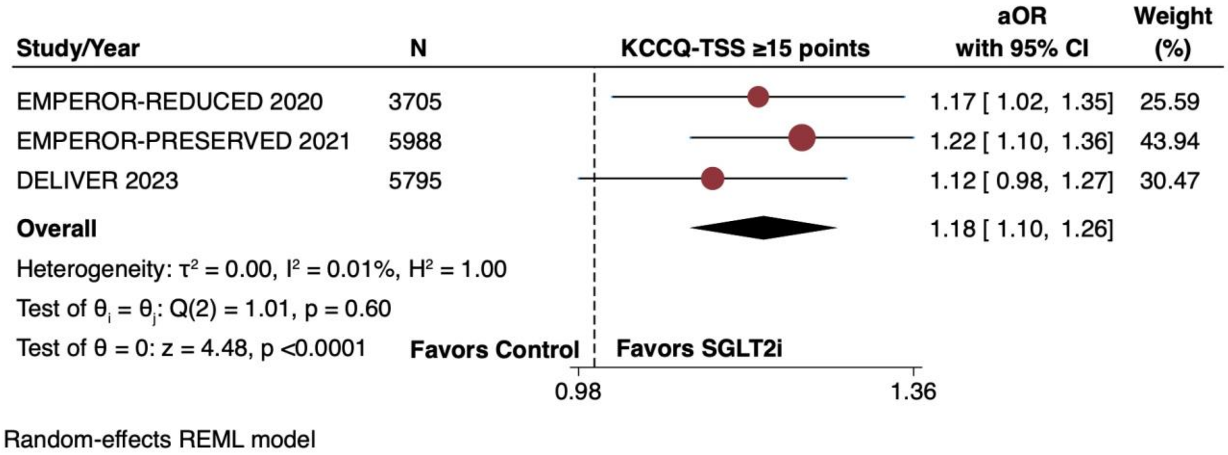

© 2024 Gao M et al. *JAMA Network Open.*

G. Improvement in KCCQ-CSS Scores by ≥ 5 Points

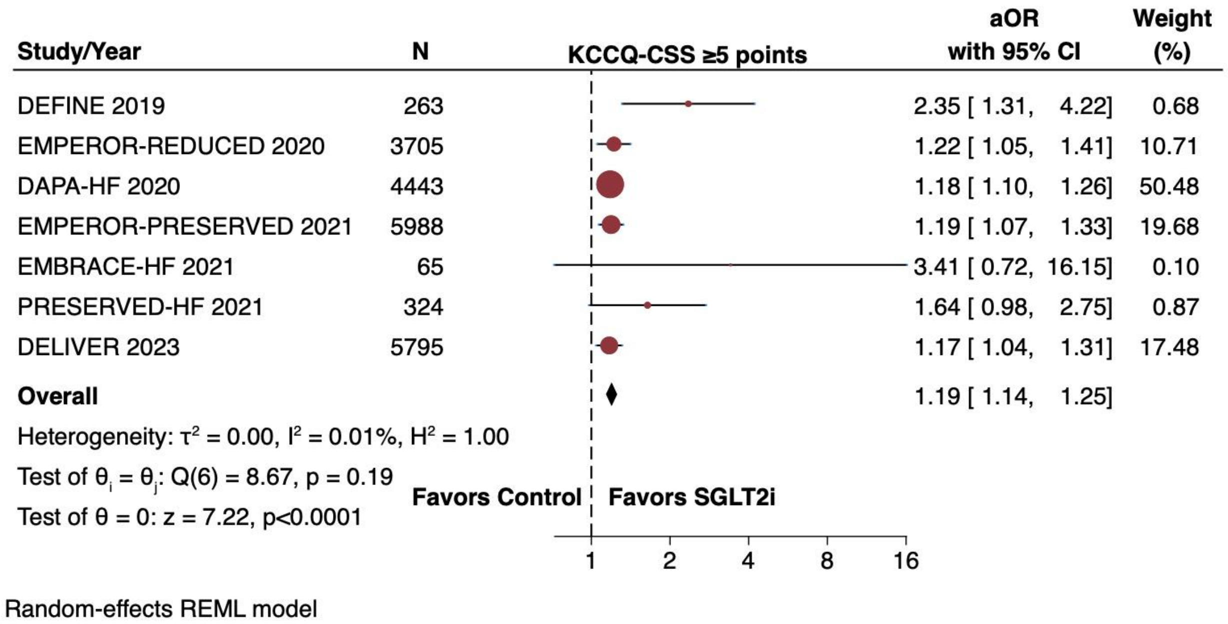

H. Improvement in KCCQ-CSS Scores by ≥ 10 Points

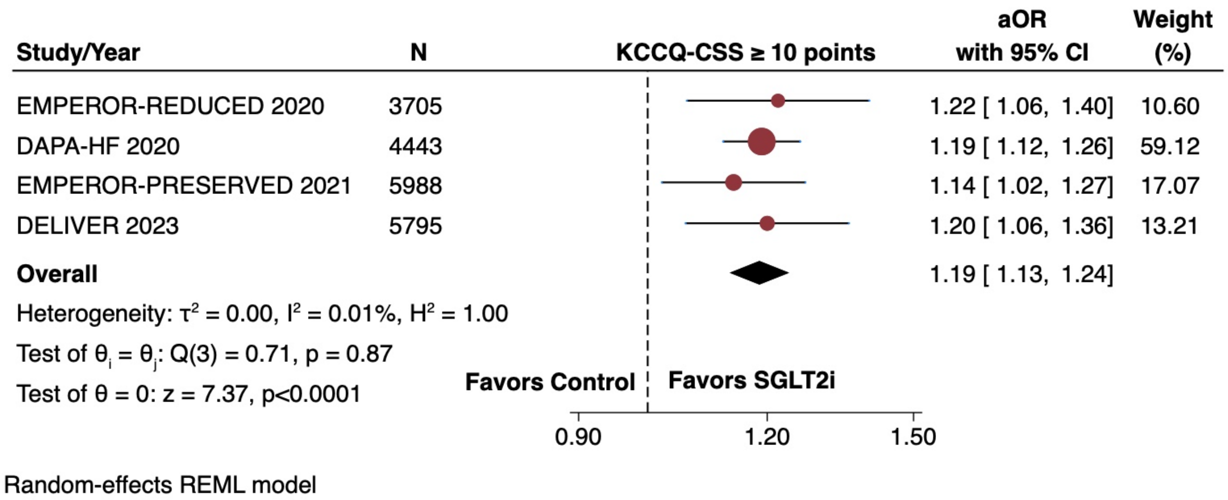

© 2024 Gao M et al. *JAMA Network Open.*

I. Improvement in KCCQ-CSS Scores by ≥ 15 Points

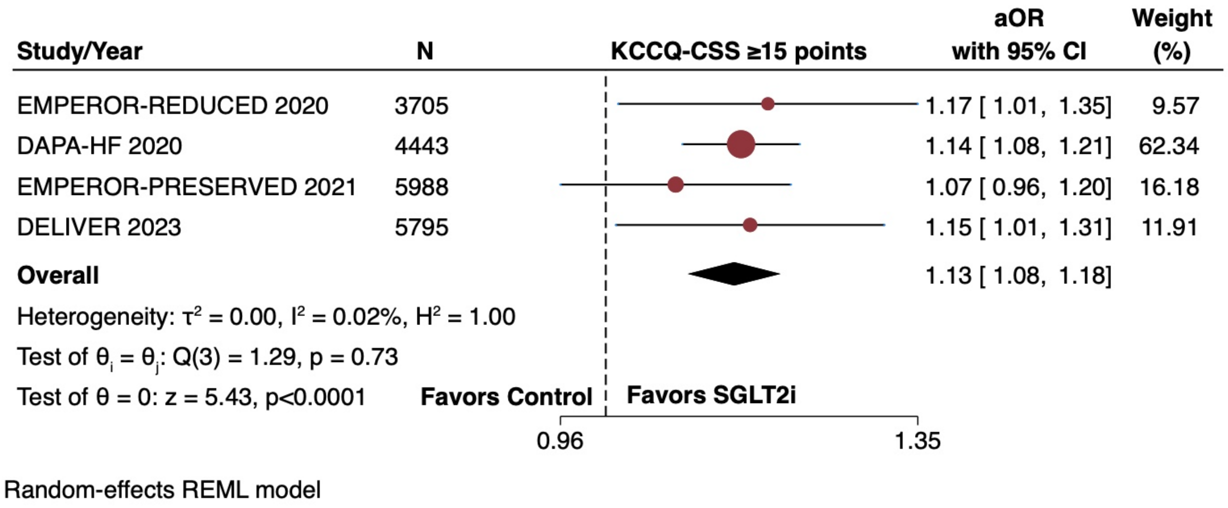

© 2024 Gao M et al. *JAMA Network Open.*

eFigure 8. Bubble Plots by Proportion of Females and Diabetic Patients Included in Trials Reporting KCCQ-TSS, KCCQ-OS, and KCCQ-CSS

A. Bubble Plot for KCCQ-TSS and Proportion of Females Included in Each Trial

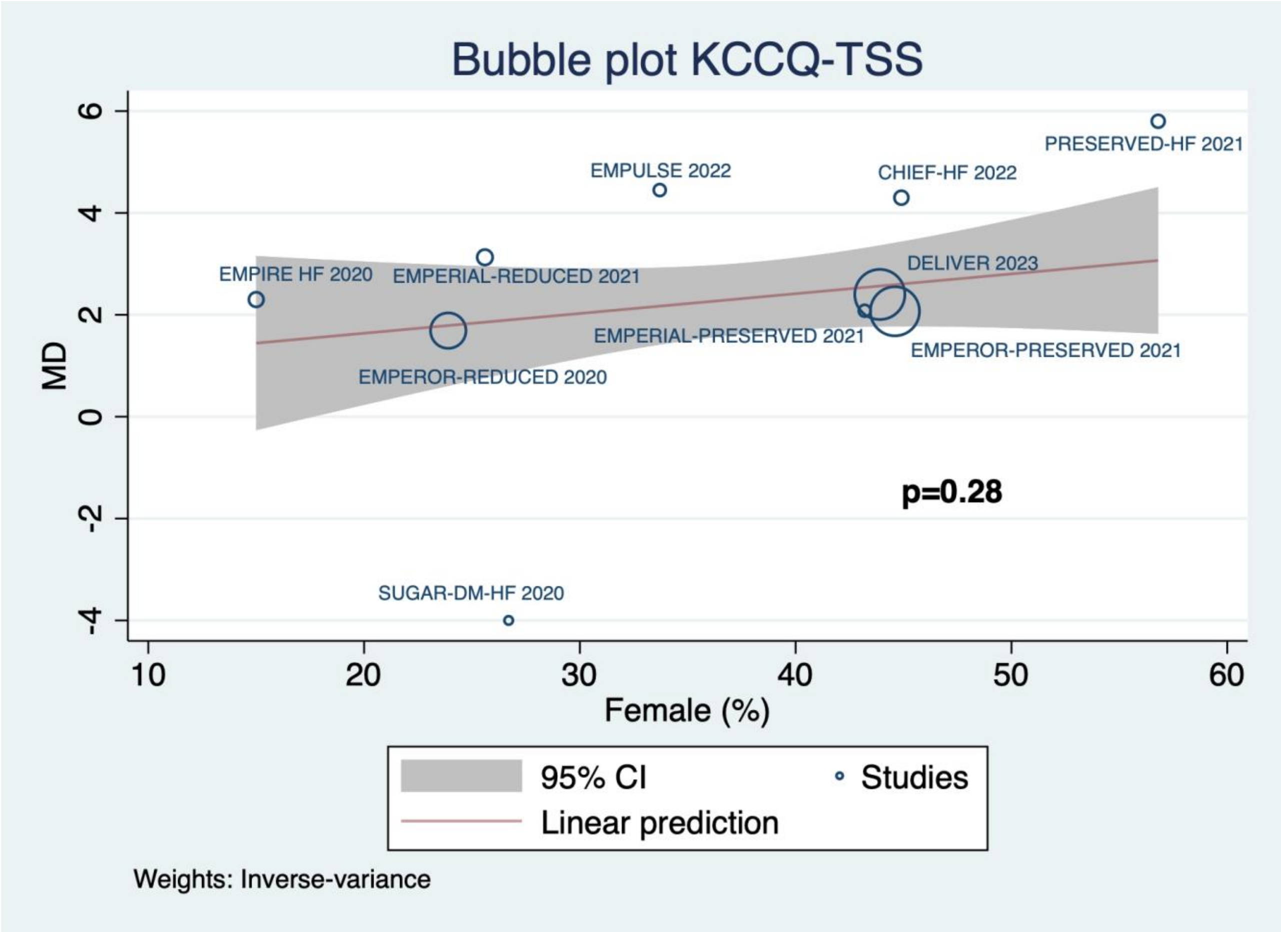

© 2024 Gao M et al. *JAMA Network Open.*

B. Bubble Plot for KCCQ-TSS, and Proportion of Type 2 Diabetic Patients Included in Each Trial

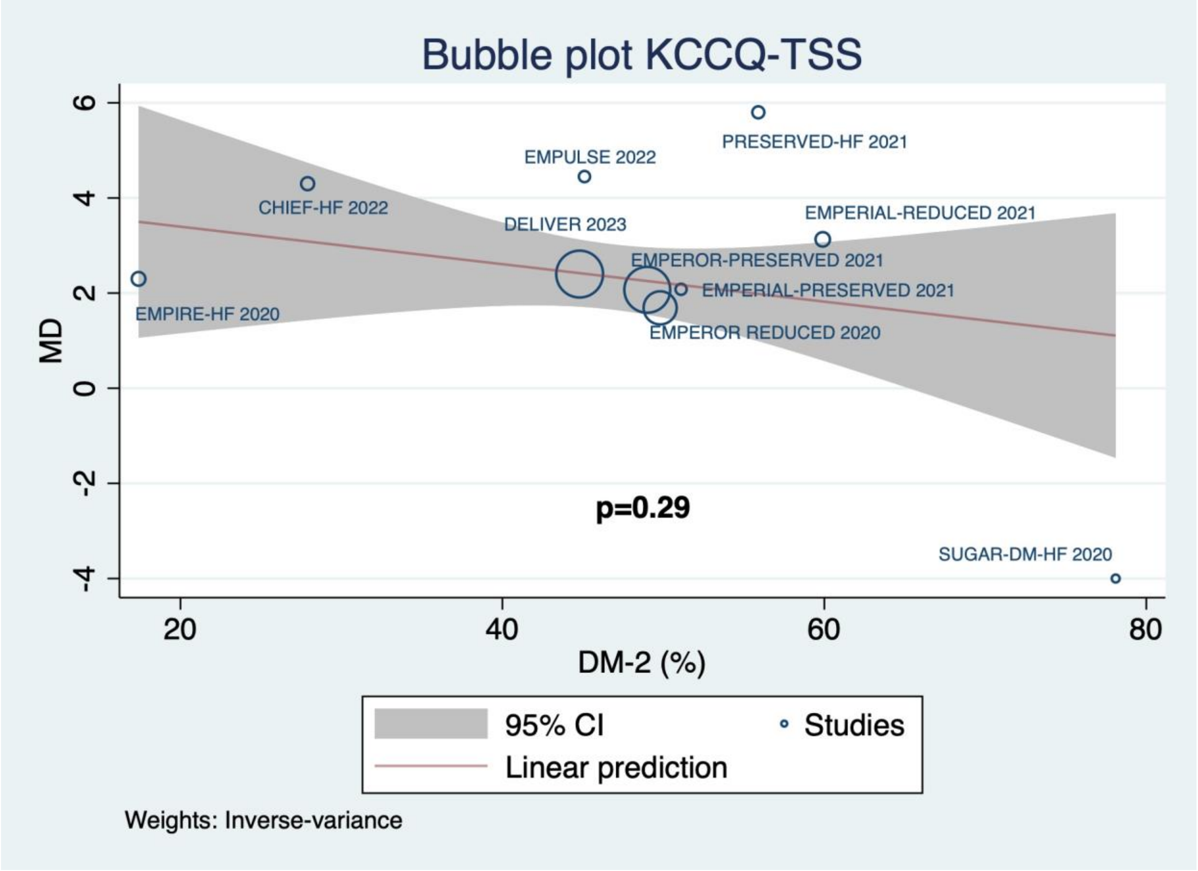

© 2024 Gao M et al. *JAMA Network Open.*

C. Bubble Plot for KCCQ-CSS and Proportion of Females Included in Each Trial

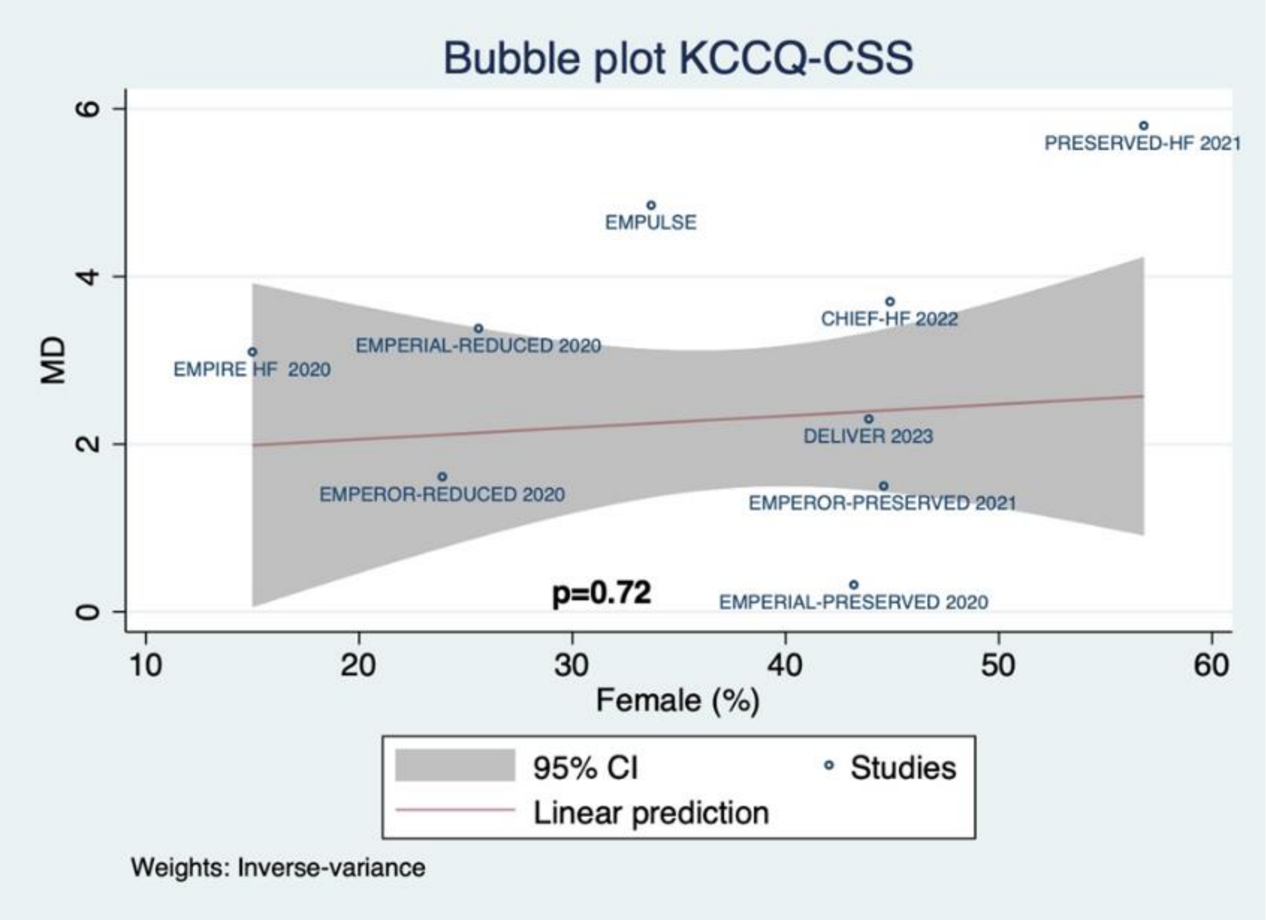

© 2024 Gao M et al. *JAMA Network Open.*

D. Bubble Plot for KCCQ-CSS, and Proportion of Type 2 Diabetic Patients Included in Each Trial

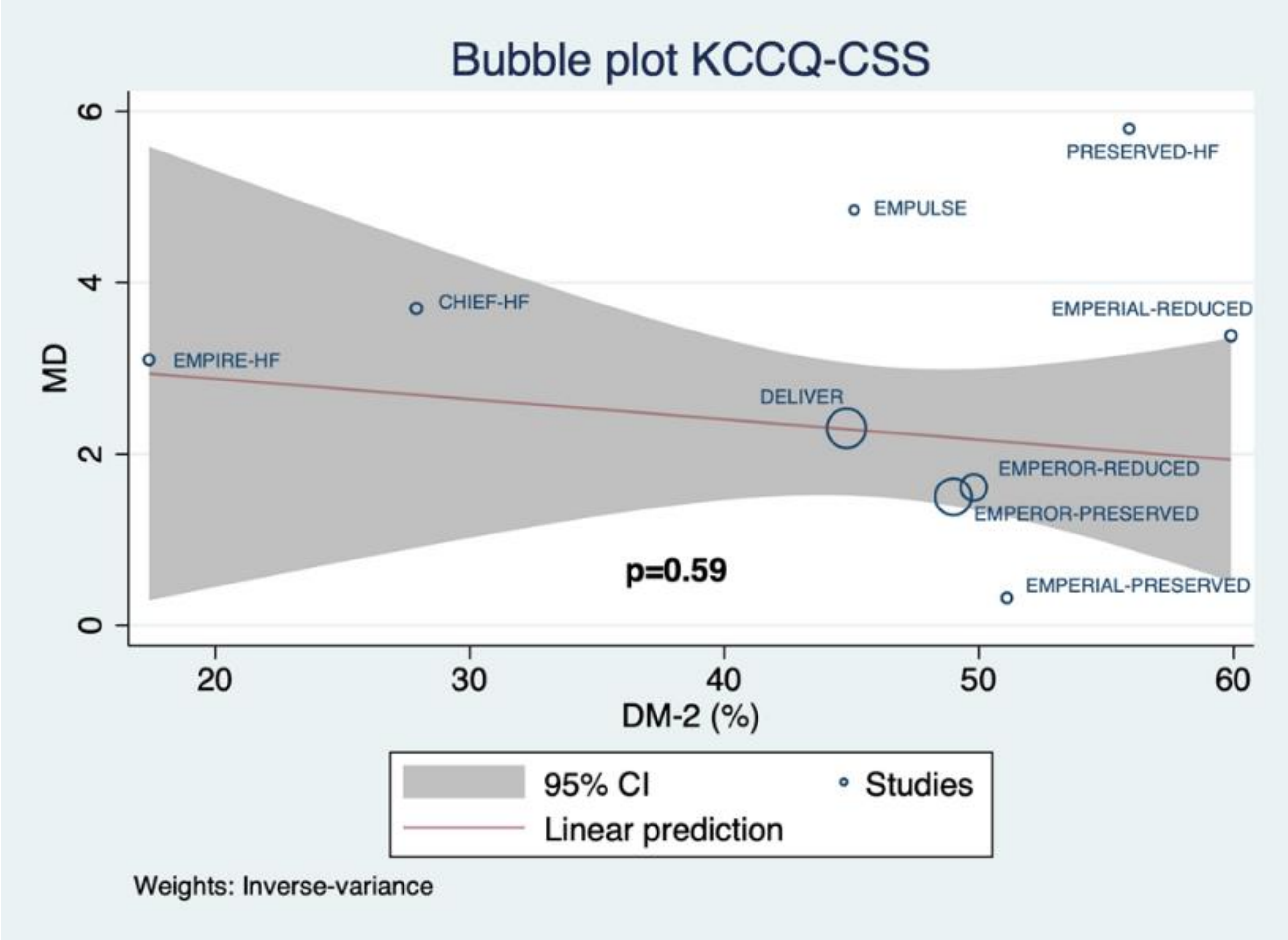

© 2024 Gao M et al. *JAMA Network Open.*

E. Bubble Plot for KCCQ-OS and Proportion of Females Included in Each Trial

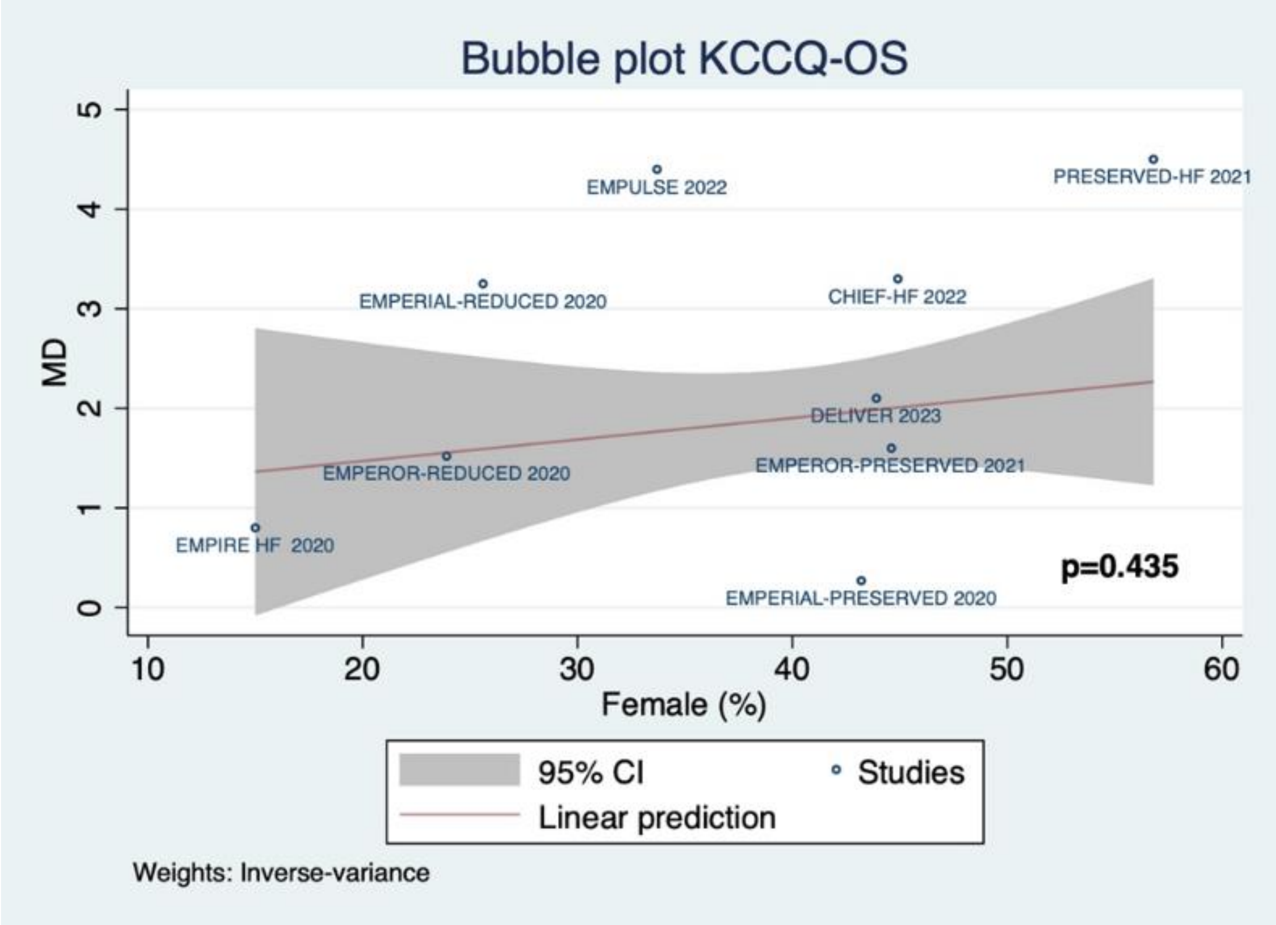

© 2024 Gao M et al. *JAMA Network Open.*

F. Bubble Plot for KCCQ-OS, and Proportion of Type 2 Diabetic Patients Included in Each Trial

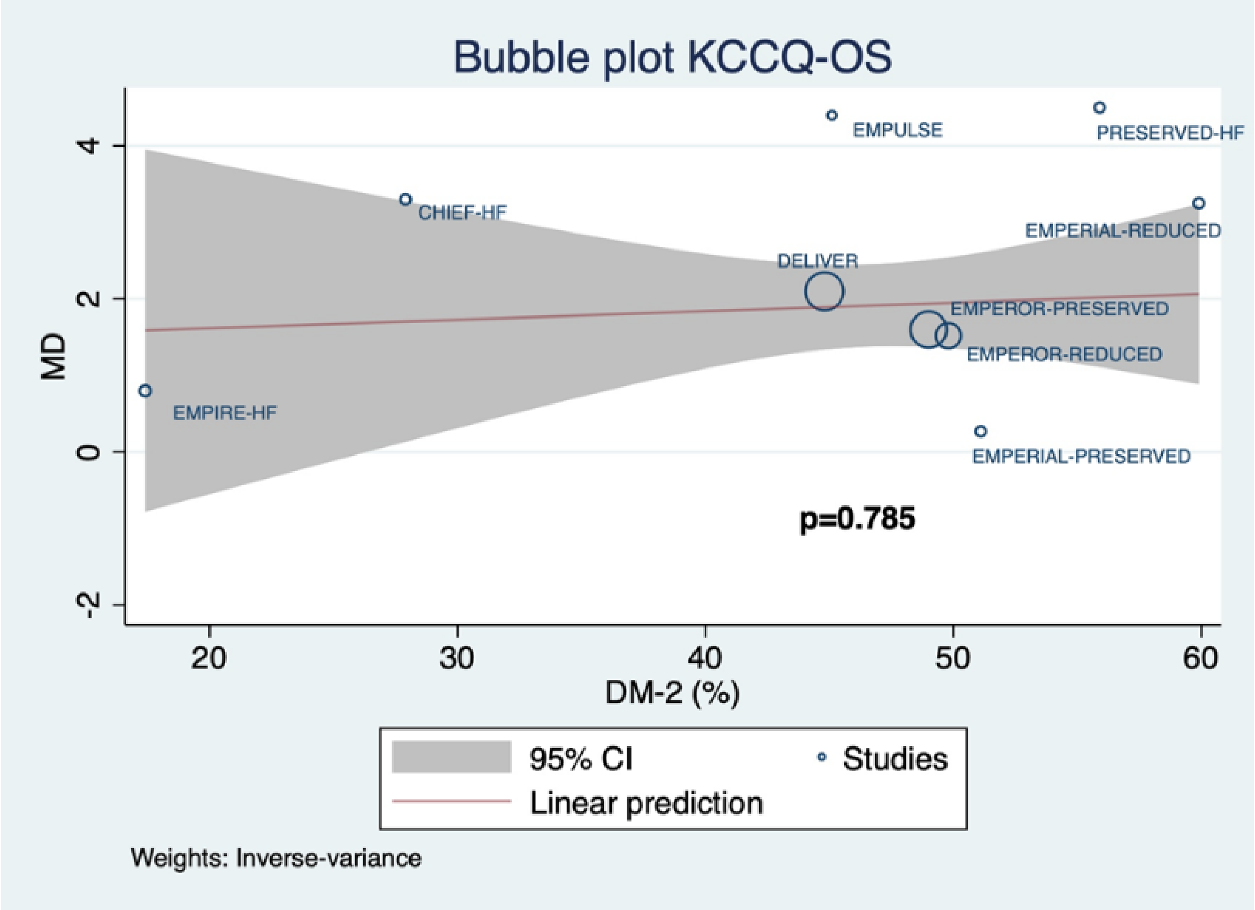

© 2024 Gao M et al. *JAMA Network Open.*

eFigure 9. Funnel Plot: KCCQ-TSS

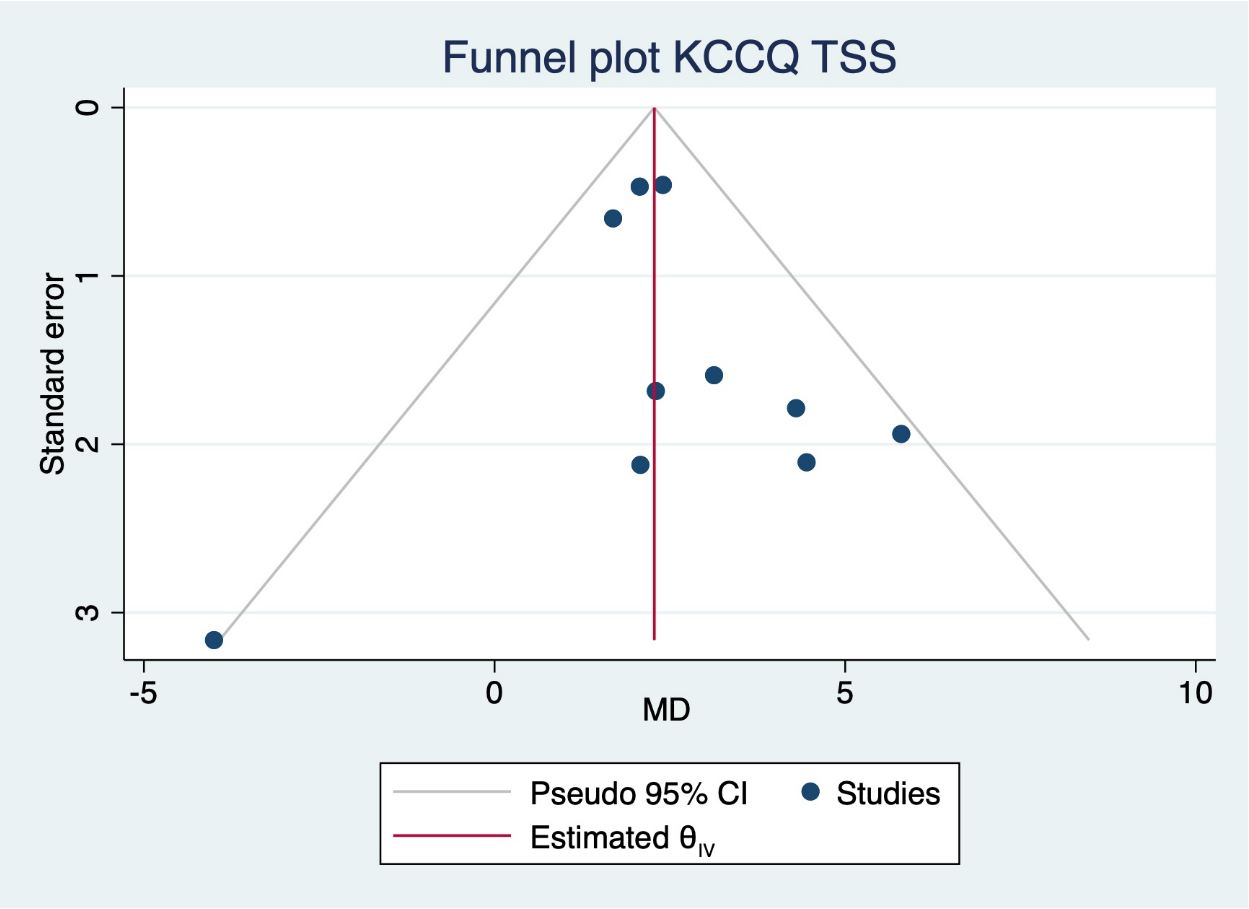

© 2024 Gao M et al. *JAMA Network Open.*
