# Supplementary material for: SGLT2 Inhibitors, Functional Capacity, and Quality of Life in Patients With Heart Failure: A Systematic Review and Meta-Analysis
Source: JAMA Netw Open. 2024 Apr 4;7(4):e245135. doi: 10.1001/jamanetworkopen.2024.5135 (PMC11192183; doi:10.1001/jamanetworkopen.2024.5135)
Supplement: Supplement 2. — Data Sharing Statement [file jamanetwopen-e245135-s002.pdf]

Data Sharing Statement

Gao. SGLT2 Inhibitors, Functional Capacity, and Quality of Life in Patients with Heart Failure. *JAMA Netw Open*. Published April 04, 2024. doi:10.1001/jamanetworkopen.2024.5135

Data
Data available: Yes
Data types: Data (not involving human participants)
How to access data: anu.lala@mountsinai.org
When available: With publication

Supporting Documents
Document types: None

Additional Information
Who can access the data: Anyone requesting data whose identity, role, and institution are verified
Types of analyses: For any purpose
Mechanisms of data availability: With investigator support and approval
Any additional restrictions: None
